# Resolution of tissue signatures of therapy response in patients with recurrent GBM treated with neoadjuvant anti-PD1

**DOI:** 10.1038/s41467-021-24293-4

**Published:** 2021-06-29

**Authors:** Yue Lu, Alphonsus H. C. Ng, Frances E. Chow, Richard G. Everson, Beth A. Helmink, Michael T. Tetzlaff, Rohit Thakur, Jennifer A. Wargo, Timothy F. Cloughesy, Robert M. Prins, James R. Heath

**Affiliations:** 1 Institute for Systems Biology, Seattle, WA USA; 2 Department of Medical and Molecular Pharmacology, David Geffen School of Medicine, University of California, Los Angeles, Los Angeles, CA USA; 3 Department of Surgical Oncology, The University of Texas MD Anderson Cancer Center, Houston, TX USA

**Keywords:** Cancer microenvironment, Immunology, Systems biology

## Abstract

The response of patients with recurrent glioblastoma multiforme to neoadjuvant immune checkpoint blockade has been challenging to interpret due to the inter-patient and intra-tumor heterogeneity. We report on a comparative analysis of tumor tissues collected from patients with recurrent glioblastoma and high-risk melanoma, both treated with neoadjuvant checkpoint blockade. We develop a framework that uses multiplex spatial protein profiling, machine learning-based image analysis, and data-driven computational models to investigate the pathophysiological and molecular factors within the tumor microenvironment that influence treatment response. Using melanoma to guide the interpretation of glioblastoma analyses, we interrogate the protein expression in microscopic compartments of tumors, and determine the correlates of cytotoxic CD8+ T cells, tumor growth, treatment response, and immune cell-cell interaction. This work reveals similarities shared between glioblastoma and melanoma, immunosuppressive factors that are unique to the glioblastoma microenvironment, and potential co-targets for enhancing the efficacy of neoadjuvant immune checkpoint blockade.

## Abstract (web summary)

The response to neoadjuvant immune checkpoint blockade (ICB) in patients with recurrent gliolastoma multiforme (GBM) has been challenging to interpret. Here the authors develop a tumor analysis framework that reveals molecular similarities between GBM and melanoma and unique patterns of immunosuppression in GBM indicating potential co-targets for neoadjuvant ICB.

## Introduction

Glioblastoma multiforme (GBM) is a primary malignancy of the central nervous system, with an incidence of 3.19 per 100,000 and a three-year survival rate of around 10%^1^. The molecular heterogeneity of GBM, its resistance to standard therapies, and immune evasion^2–4^ all contribute towards GBM pathogenesis. By comparison, although clinical outcomes for patients with advanced melanomas have historically been poor^5^, advances in adjuvant checkpoint immunotherapies, and especially combination (anti-PD1 + anti-CTLA4) immunotherapies, have demonstrated significant patient survival benefits^6^, to the extent that melanoma has become a model cancer for understanding immunotherapy responses.

Recent reports of checkpoint immunotherapies in the neoadjuvant setting suggest that such a regimen may ultimately yield additional benefits^7,8^. For example, in a recent small randomized trial for patients with high-risk resectable melanoma^9^, neoadjuvant anti-PD1 monotherapy yielded modest response rates (overall response rate [ORR] 25%, pathological complete response [pCR] 25%) and low toxicity (8% grade 3 treatment-related adverse events [trAEs]), while neoadjuvant anti-PD1 plus anti-CTLA-4 combination therapy yielded higher response rate (ORR 73%, pCR 45%), but substantial toxicity (73% grade 3 trAEs). In either therapy, immune correlates of response were identified, suggesting that an optimized treatment regimen of neoadjuvant checkpoint blockade may yield strong patient benefit with reduced toxicity in high-risk melanoma. Similarly, recent reports by some of us^10^ and others^11–13^ suggest that neoadjuvant anti-PD1 therapy for patients with recurrent GBM can promote a survival benefit. For the GBM study, neoadjuvant and adjuvant anti-PD1 therapy (pembrolizumab) were tested in a randomized, multi-institution clinical trial^10^. Parallel to the neoadjuvant immunotherapy trial in melanoma^9^, elevated immune correlates of response were detected in resected tumors, but were largely limited to the patients in the neoadjuvant treatment arm. The effectiveness of neoadjuvant checkpoint blockade should be reflected in the pathophysiological and molecular features presented in the tumor, but the exact nature of those features will exhibit similarities and differences across tumor settings, and from patient-to-patient, and so may be challenging to interpret.

**Fig. 1:**
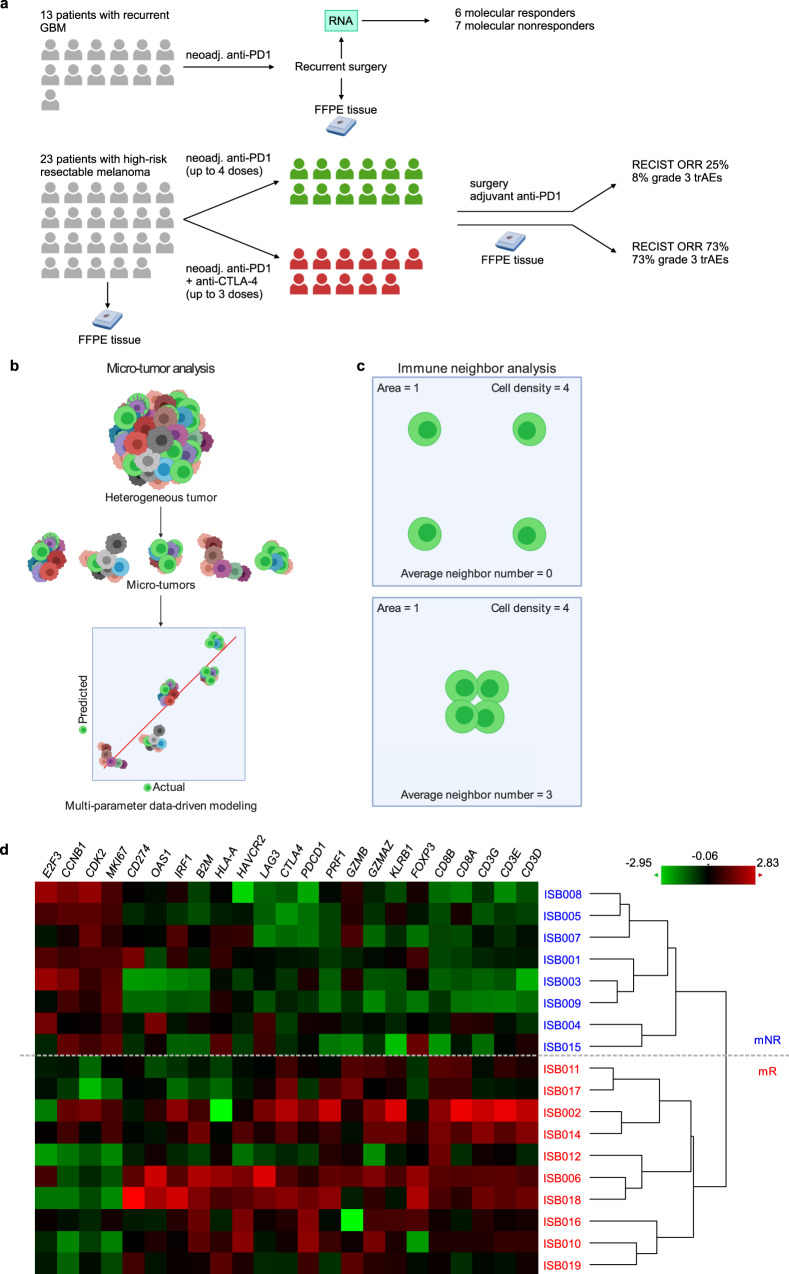
Study schema and specimens collected. **a** Top panel: Patients with recurrent GBM were treated with off-label neoadjuvant anti-PD1 ~14 days before surgical resection. Tumors were collected for bulk RNA analysis and FFPE sections. Bottom panel: Patients with high-risk resectable melanoma were enrolled in a randomized phase 2 trial (NCT02519322). Tumors were collected at baseline and on-treatment for FFPE sections. Patients were randomized to receive neoadjuvant nivolumab (green) or ipilimumab plus nivolumab (red). **b** Scheme of micro-tumor analysis. A heterogeneous tumor is analyzed in smaller parts (micro-tumors), where each micro-tumor is deeply characterized and has its own local therapy response (e.g., abundance of T cells). These micro-tumors are used to build a model, which can reveal the important variables that determine the response. **c** Scheme of immune neighbor analysis. Immune cell–cell interaction is quantified by counting the number of adjacent immune-cell neighbors for each immune cell. The same density of cells can yield different average neighbor numbers. **d** Molecular response of neoadjuvant anti-PD1 treatment in recurrent GBM determined by bulk gene signatures. The genes and samples were arranged with hierarchical clustering. mNR molecular nonresponder, mR molecular responder, FFPE formalin-fixed paraffin-embedded, RECIST response evaluation criteria in solid tumors, ORR overall response rate, trAEs treatment-related adverse events. Green coloration represents low expression; red coloration represents high expression. Schemes in (**a**), (**b**), and (**c**) were created with BioRender.com. Source data are provided as a Source Data file.

Here, we report on an in-depth, spatially resolved comparative analysis of tissues collected from patients with recurrent GBM and high-risk melanoma, both treated with neoadjuvant immune checkpoint blockade (ICB) (Fig. 1a). We utilize the melanoma tumor analyses to guide the interpretation of the GBM tissues by making three distinct sets of comparisons. First, we compare the levels of a protein panel collected from microscopic compartments within both classes of tumors^14^. Second, we develop an approach called micro-tumor analysis (Fig. 1b) to computationally determine the correlates of cytotoxic CD8+ T cells and treatment response in both tumors, as well as tumor growth in GBM. Third, we develop a method called immune neighbor analysis (Fig. 1c) to map out the immune cells in both tumors and compare their degree of immune cell–cell interaction, which was defined by the number of immune cells surrounding any specific immune cell, averaged across the whole tissue section. Broadly, we present a framework to uncover pathophysiological and molecular features that determine the effectiveness of immunotherapies.

## Results

### Characteristics of patients with recurrent GBM and high-risk melanoma

In a recent multi-institution clinical trial (NCT02852655), we showed that patients with recurrent GBM, who were randomized to receive neoadjuvant pembrolizumab (pembro) with continued post-surgical adjuvant therapy, had significantly extended overall survival (OS) compared to patients who were randomized to receive post-surgical adjuvant pembro alone^10^. Bulk RNA sequencing of the tumors revealed that neoadjuvant pembro treatment was associated with upregulation of T cell and interferon-gamma (IFN-γ) genes, and downregulation of cell cycle genes. This analysis yielded a 23-gene signature that was associated with longer OS and progression-free survival (PFS) as determined by a modified response assessment in neuro-oncology (RANO) criteria^15^, making it a useful molecular predictor of clinical response.

To study the pathophysiological and molecular factors of therapy response at a higher resolution, we examined tumor samples collected from 13 follow-on patients with recurrent GBM, who received anti-PD1 off label 14 ± 5 days before surgical resection (Fig. 1a, top and Supplementary Table 1). Two patients had successive recurrences and were pre-treated with pembro before each surgery. One patient had multifocal tumor, and specimens were collected from the original tumor location and at a secondary site (Supplementary Table 1). In total 18 tumor samples were collected.

Unlike the original clinical trial, where patients were heavily screened for eligibility to ensure uniform clinical characteristics (i.e., first or second recurrence, similar tumor size, and low corticosteroid treatment levels), the follow-on patients here have varying clinical characteristic (Supplementary Table 1), precluding the direct comparison of their RANO clinical outcomes. Therefore, to categorize the therapeutic response, we analyzed the resected tumors by targeted mRNA analysis, specifically looking at the 23-gene molecular response signature of the original trial. Hierarchal clustering of the tumors using this gene signature yielded a cluster characterized by high T cell and IFN-γ and low cell cycle gene expression (Fig. 1d). We defined this group as the molecular responder (mR) group. Tumors in a second cluster with the opposite signature were defined as the molecular nonresponder (mNR) group.

As a reference, we also examined baseline and on-treatment melanoma tumors from 23 patients with high-risk resectable melanoma, who were enrolled in a randomized phase 2 study of neoadjuvant nivolumab (nivo) versus combined ipilmumab with nivolumab (ipi-nivo) (Fig. 1a, bottom and Supplementary Table 2)^9^. The patients were categorized as responder (R) or nonresponder (NR) based on the response evaluation criteria in solid tumors (RECIST) criteria, with higher lymphoid infiltrates observed in responders to both therapies.

### Proliferation signature is localized to the tumor compartments of GBM

**Fig. 2:**
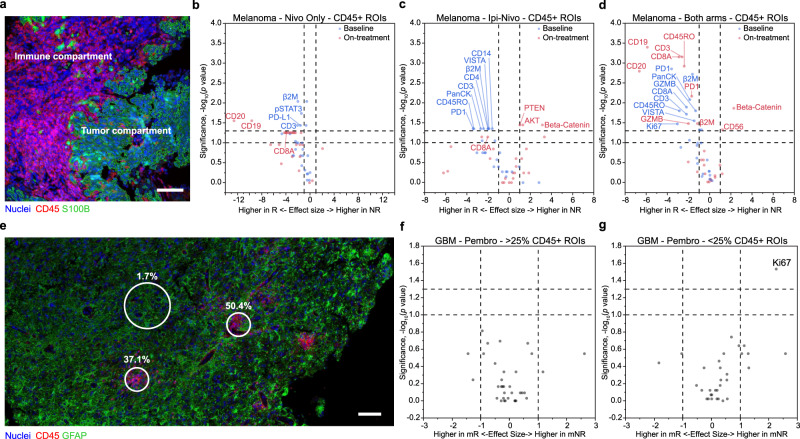
Regions of interest definition and differential protein expression. **a** Fluorescent micrograph of a representative region in a melanoma sample (*n* = 39) stained for nuclei (blue), CD45 (red), and S100B (green). The immune compartment (contoured with red lines) and the tumor compartment (contoured with green lines) were identified via thresholding the fluorescence intensity of CD45 marker. Scale bar is 100 µm. **b**–**d** Volcano plots of two-sided Mann–Whitney *U*-test comparisons of the protein expressions between RECIST responders and nonresponders in baseline (blue) and on-treatment (red) melanoma samples. The samples were either from the nivolumab (nivo) cohort (**b**) (*n* = 9 nonresponders and 3 responders at baseline, *n* = 7 nonresponders and 2 responders on-treatment), ipilimumab plus nivolumab (ipi-nivo) cohort (**c**) (*n* = 2 nonresponders and 8 responders at baseline, *n* = 3 nonresponders and 5 responders on-treatment), or both arms combined (**d**) (*n* = 11 nonresponders and 11 responders at baseline, *n* = 10 nonresponders and 7 responders on-treatment). Expression of proteins was assessed in the immune compartments of the tissue and quantified as the average count per area of assayed tissue. **e** Fluorescent micrograph of a representative region in a GBM sample (*n* = 14) stained for nuclei (blue), CD45 (red), and GFAP (green). Three representative geometric ROIs (in white) were designated as immune-cell rich (immune cell proportion: 37.1% and 50.4%) and immune-cell poor (immune cell proportion: 1.7%). Scale bar is 100 µm. **f**, **g** Volcano plots of two-sided Mann–Whitney *U*-test comparisons of the protein expressions between molecular responders (mR) and molecular nonresponders (mNR) in GBM samples treated with pembrolizumab (pembro). The responses were generated based on a 23-gene molecular signature. Expression of proteins was quantified as average count per area of assayed tissue, which were either in the immune-cell rich regions (**f**) (*n* = 8 mNRs and 4 mRs) or immune-cell poor regions (**g**) (*n* = 8 mNRs and 6 mRs). For plots in (**b**–**d**), (**f**), and (**g**), the dashed horizontal lines represent the *p* value cutoffs (*p* < 0.10 and *p* < 0.05) and the dashed vertical lines represent the effect size cutoffs (effect size > |1|). ROIs regions of interest. Source data are provided as a Source Data file.

We profiled melanoma and GBM tissues using a spatially resolved multiplexed protein analysis approach^16^, characterizing the expression of up to 40 immuno-oncology (IO) proteins in formalin-fixed paraffin-embedded (FFPE) tissue slices (Fig. 2). Including controls, 31 IO proteins were measured in both tumors, two proteins were measured only in melanoma, and 11 proteins were measured only in GBM (Supplementary Table 3).

In both tumors, CD45 was employed as a visualization marker to identify microscopic immune regions of interest (ROIs). For melanoma, ROIs were defined by a manually set signal threshold from fluorescent anti-CD45 to create a custom mask that delineates the immune and tumor compartments (Fig. 2a). We defined up to seven custom ROIs per tissue, with a focus on the immune-cell-rich compartments. Overall, we analyzed 208 ROIs across 39 melanoma tumors, of which 10 are baseline NR, 11 are baseline R, 10 are on-treatment NR, and 8 are on-treatment R (Supplementary Table 2). In the immune compartments of melanoma tissues, proteins implicated in immune response were enriched in the responders after treatment (Fig. 2b–d), including CD8A, CD19/CD20, and CD45RO. This indicates higher lymphoid infiltration and activation in responders, consistent with the previous analyses^9^.

GBM tumors are different from melanoma tumors in terms of size and immune infiltration, warranting a different ROI approach. The GBM tumors are both much larger in cross section (10–30 times), and have far fewer (and smaller) immune regions that can be far apart. Applying the custom-masked ROI strategy on GBM would have required a prohibitively large number of ROIs, which would only sample a very small portion of the tumor after CD45 threshold is applied. Thus, we used an alternative approach designed to provide a better representative sampling of the GBM tumors. We defined up to 16 geometric ROIs per tissue (3–4 times more than melanoma), choosing representative areas that were immune-cell rich and immune-cell poor across the entire tumor (Fig. 2e). We used fluorescent anti-CD45 staining to guide the identification of these areas, without masking for CD45. Furthermore, we used image analysis to quantify the proportion of immune cells (CD45+) for each ROI and defined >25% CD45+ as immune rich and <25% CD45+ as immune poor via hierarchal clustering (Supplementary Fig. 1a–c). Three representative ROIs are shown (Fig. 2e), two are immune rich (37.1% and 50.4%) and one is immune poor (1.7%). Spatial protein profiling was performed on 14 of the 18 resected GBM tumors, 6 were mRs and 8 were mNRs (Supplementary Table 1). Overall, a total of 168 ROIs were analyzed, of which 55 were immune rich and 113 were immune poor. As expected, immune-related proteins, including CD45RO, CD20, CD4, CD8A, CD68, CD11c, ICOS, and STING are higher in the immune-rich ROIs relative to the immune-poor ROIs (Supplementary Fig. 1d).

In contrast to the analysis of melanoma tumors, we did not identify proteins that differentiated mRs from mNRs in the immune-rich regions of GBM (Fig. 2f). However, we did observe higher Ki67 in the immune-poor regions for molecular nonresponders (*p* = 0.03), suggesting that the tumor was proliferating in those patients (Fig. 2g). This observation is consistent with the bulk transcriptional signature, which exhibits upregulation of cell cycle genes in mNRs (Fig. 1d). Here, we could definitively localize this proliferation signature to the tumor-dominant compartments of the tissue.

This analysis points to some stark differences between melanomas and GBMs treated with neoadjuvant ICB. First, the melanoma tumors, whether treated with nivo only or ipi-nivo, generally exhibited high levels of immune cell infiltration, and the average levels of several proteins in the immune-rich tumor compartments correlated with therapy response. The GBM tumors, by contrast, are characterized by fewer immune cell infiltrates, and those infiltrates reside in clusters of just a few cells. Furthermore, the only protein level that correlated with the mNR classification was the proliferation marker, Ki67, and that was elevated in the immune-cell poor regions of the tumors. These results demonstrate that the effect of neoadjuvant pembro in recurrent GBM is difficult to resolve, warranting a deeper analytical approach.

### Micro-tumor analysis reveals proteins that correlate with CD8 presence

To overcome inter-patient and intra-tumor heterogeneity and extract additional biological insights of treatment response, we developed a method called micro-tumor analysis, which treats each ROI as an independent microscopic tumor, each presenting its own local therapy response (Fig. 1b). We first hypothesized that a positive response to checkpoint inhibitor therapy, for either melanoma or GBM, would be reflected by CD8+ T cell infiltrates. To this end, we applied a multivariate modeling approach, called partial least squares regression (PLSR)^17–21^, with the goal of determining the correlates of CD8 presence in both melanoma and GBM. PLSR is designed to extract linear relationships between a matrix **X** of independent variables and a matrix **Y** of dependent variables through the formation of PLS components and dimensional reduction. Here, the original data is transformed into new dimensions that are linear combinations of the original variables, similar to principal component analysis, but in a way that maximizes the covariation of **X** with **Y**. Thus, the PLS components derived from this approach will provide some percentage of explanation of the variation in **Y**, and reveal which variables in **X** are most important. The orthogonality of the PLS components ensures that there are no issues of multicollinearity in the model. Notably, this analysis yields an unbiased comparison between the two tumor types.

**Fig. 3:**
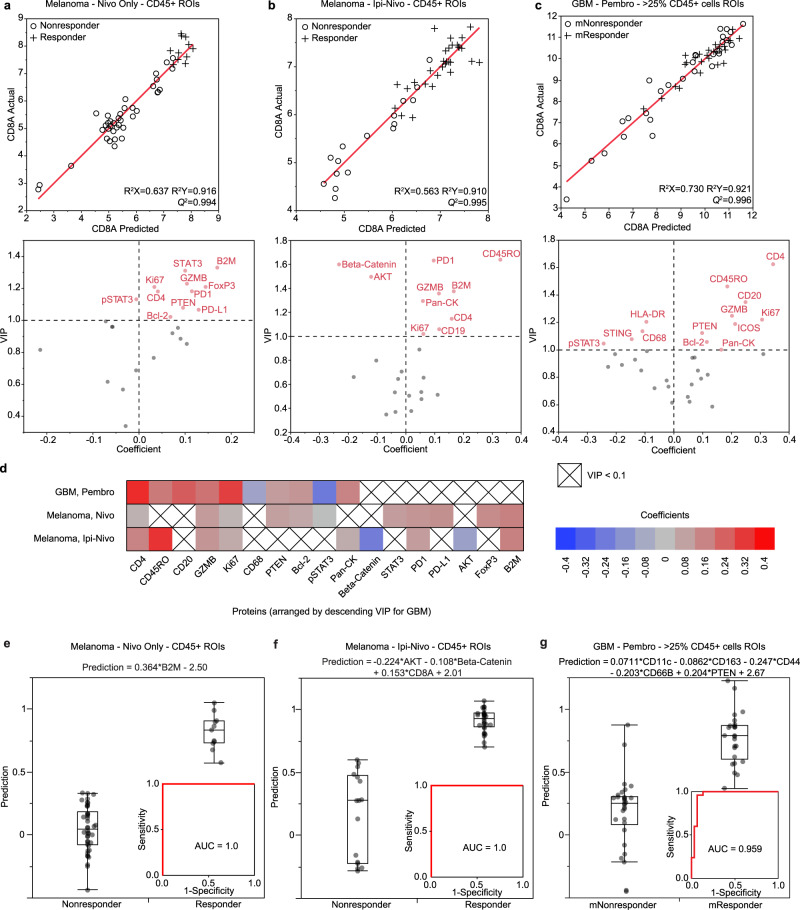
Micro-tumor analyses reveal proteins that correlate with CD8 presence and treatment response. **a**–**c** PLS regression analysis of immune-rich regions of interest with CD8A as output in melanoma samples treated with nivolumab (nivo) (**a**), melanoma samples treated with ipilimumab plus nivolumab (ipi-nivo) (**b**), and GBM samples treated with pembrolizumab (pembro) (**c**). Top panels: plots of actual observations versus model predictions of CD8A expression. Bottom panels: plots of variable importance in the projection (VIP) versus model coefficients. The dashed horizontal line represents the VIP > 1.0 cutoff and the dashed vertical line is where the model coefficient is 0. **d** Heat map summarizing the important (VIP > 1.0) CD8A predictors that were measured in both melanoma and GBM. Red coloration represents positive coefficients, and blue coloration represents negative coefficients. **e**–**g** PLS discriminant analysis of immune-rich regions of interest with treatment response as output in melanoma samples treated with nivolumab (**e**), melanoma samples treated with ipilimumab plus nivolumab (**f**), and GBM samples treated with pembrolizumab (**g**). The predictors of lower importance (VIP < 0.8, coefficient < |0.1|) were iteratively removed to determine the minimum set of predictors that enables accurate (AUC > 0.90) classification of RECIST or molecular responder and nonresponder ROIs. Shown are the model prediction equations and box plots of the final prediction model versus treatment response for individual ROIs. The horizontal line in each box represents the median sample value, the ends of the box represent the 25th and 75th percentiles, and the whiskers extend from the ends of the box to the outer most data points. Inset, the area under the receiver operating characteristics curve (AUC). ROIs regions of interest. Response for melanoma and GBM are based on RECIST criteria and a 23-gene molecular signature, respectively. Source data are provided as a Source Data file.

We applied PLSR on the immune-rich compartments of the on-treatment tumors using CD8 as the dependent variable (**Y**) and the other measured proteins as independent variables in **X** to yield predictions of CD8 presence (Fig. 3a–d). In the analysis of melanoma samples treated with nivo, melanoma samples treated with ipi-nivo, and GBM samples treated with pembro, the optimal number of PLS components are 3, 4, and 6, respectively (Supplementary Fig. 2a–c). Plots of actual observations versus model predictions demonstrate that the models accurately capture the variance in the inputs (R^2^X) and outputs (R^2^Y), and have excellent predictability (*Q*^2^ > 0.99) (Fig. 3a–c, top panels). In the two melanoma plots, the responder ROIs tend to cluster in the high CD8 region (top right), while nonresponder ROIs tend to cluster in the low CD8 region (bottom left). This is consistent with the differential analysis described above (Fig. 2b–d) and in the previous studies^22^. In the case of GBM, the molecular responder ROIs also tend to cluster in the high CD8 region (top right), but the molecular nonresponder ROIs are found across the full spectrum of CD8 levels.

To determine the important variables in **X**, we looked at the variable importance of projection (VIP) score for each variable, which is a weighted sum of the contributions of that variable, from all the PLS components, toward predicting CD8 presence. Plots of VIP versus model coefficients reveal the important proteins (VIP > 1) in the model, and whether they are negatively (−ve coefficient) or positively (+ve coefficient) correlated with CD8 (Fig. 3a–c, bottom panels). For proteins measured only in GBM, we observed that ICOS is positively correlated with CD8, while HLA-DR and STING are negatively correlated with CD8 (Fig. 3c, bottom panel). Of the proteins that were measured across all three tumor datasets, we observed that CD4, CD45RO, GZMB, Ki67, PTEN, Bcl-2, CD19/20, and Pan-CK are positively correlated with CD8 in GBM and in at least one melanoma treatment arm (Fig. 3d). In addition, we observed that pSTAT3 is negatively correlated with CD8 in GBM and, to a much smaller degree, in melanoma (nivo). On the other hand, we observed that β2M and PD1 are positively correlated with CD8 only in melanoma, while the macrophage marker CD68 is negatively correlated with CD8 only in GBM.

To test the utility of the PLSR approach, we compared its performance with a simple correlation analysis for CD8 in GBM (Supplementary Fig. 2d). For 4 of the 12 important proteins (VIP > 1) identified by the PLSR analysis (Pan-CK, STING, CD68, and HLA-DR), their model coefficients had a different sign from their correlation coefficient (*r*) with CD8. Furthermore, although pSTAT3 is the most negatively correlated protein with CD8 in the PLSR model, the corresponding *r* metric suggests no correlation (−0.05). These results highlight two main advantages of using PLSR over a simple correlation analysis. First, PLSR is designed to address the problem of multicollinearity among variables. A simple correlation analysis between two variables can lead to confounding results if there is a third variable numerically related to both variables of interest. Second, PLSR provides a statistical framework (VIP) to identify variables that exhibit significant covariation with CD8. Whereas in a correlation analysis, one needs to subjectively decide on how to interpret the r metric.

To test whether the PLSR model was sensitive to changes in the 25% CD45 cutoff value, we performed the same analysis at 20% and 30% CD45 cutoff. We observed that the proteins with the highest statistical importance (leftmost proteins, VIP > 1.15) remain unchanged (Supplementary Fig. 2e), suggesting that small changes in the immune cell cutoff do not affect the results. Overall, we found that the PLSR approach is robust, and the top correlates of CD8 presence are preserved between both tumor classes, suggesting that a comparison of melanoma and GBM immune-rich ROIs could be carried out despite using different ROI strategies.

### Micro-tumor analysis reveals proteins that correlate with treatment response

From the differential analysis (Fig. 2f–g), we have shown in GBM that there is no single measured protein that can clearly discriminant mRs from mNRs. Thus, we sought to determine the minimum variables that enable such discrimination, and hypothesized that these variables may have biological significance in determining treatment outcomes. To this end, we employed partial least squares (PLS) discriminant analysis (PLS-DA), which builds a classification model using the measured proteins in **X** and a categorical variable in **Y** (i.e., RECIST or molecular responder = 1, RECIST or molecular nonresponder = 0). The classification performance of the model is evaluated by computing the area under the receiver operating characteristics (ROC) curve (AUC). To determine the minimum variables required for accurate discrimination (AUC > 0.90), we successively removed proteins of low importance (VIP < 0.8 and coefficient < |0.1|) and performed PLS-DA on the decreasing subsets of proteins.

We applied this PLS-DA workflow on the immune compartments of the on-treatment melanoma (Supplementary Fig. 3a–b). In the nivo treatment arm, we found that the most important protein is β2M, which is correlated with RECIST response and enables perfect classification of R and NR ROIs (AUC = 1.0) (Fig. 3e). In the ipi-nivo treatment arm, the most important proteins are CD8, AKT, and Beta-Catenin, which enable perfect classification of R and NR ROIs (AUC = 1.0). Here, CD8 is correlated with response, while AKT and Beta-Catenin correlate with nonresponse (Fig. 3f). PLS-DA analysis on the combined treatment arms revealed that the most important proteins are β2M, Beta-Catenin, CD19, and CD8A, which together can accurately discriminate R and NR ROIs (AUC = 0.998) (Supplementary Fig. 4a). Here, CD8, CD19, and β2M are correlated with response, while Beta-Catenin is correlated with nonresponse (Supplementary Fig. 4b, c). These results suggest that CD8, CD19, and MHC Class I antigen presentation promote treatment response, while the presence of Beta-Catenin suppresses that response. Overall, these observations recapitulate the findings in the differential analyses (Fig. 2b, d) and PLSR analyses (Fig. 3a, b, bottom panel), demonstrating the self-consistency of distinct analysis approaches.

In the PLS-DA analysis of immune-rich regions of GBM (Supplementary Fig. 5a), the most important proteins are CD11c, CD163, CD44, CD66B, and PTEN, which can classify the ROIs with high accuracy (AUC = 0.959) (Fig. 3g). Here, CD11c and PTEN are correlated with molecular response, while CD163, CD44, and CD66B are correlated with molecular nonresponse (Supplementary Fig. 5b, c). When we applied the prediction formula at the tissue level, we found that these 5 proteins can separate the tissues of mRs from mNRs (*p* = 0.03), and accurately classify those tissues (AUC = 0.907) (Supplementary Fig. 5d). These data suggest that CD11c+ cells may promote molecular response, while neutrophils (CD66B) and M2 macrophages (CD163) suppress that response. Furthermore, consistent with the correlations observed in this analysis, CD44 has been associated with cellular mobility and GBM aggressiveness^23^ and PTEN loss has been associated poor outcomes in recurrent GBM treated with neoadjuvant anti-PD1^11^.

In contrast to the immune-rich regions of GBM, PLS-DA analysis of the immune-poor regions with the full protein panel yielded modest discrimination of mR and mNR ROIs (AUC = 0.889). While further variable reduction does not improve the AUC, we found that the remaining most important proteins (Ki67, AKT, B7-H3, and CD34) can still modestly discriminant mR and mNR ROIs (AUC = 0.861) (Supplementary Fig. 6). Here, Ki67, AKT, and B7-H3 are correlated with molecular nonresponse, while CD34 is correlated with molecular response. This observation reinforces the importance of tumor proliferation as a predictor of therapy response in the tumor-dominant regions of GBM.

### Micro-tumor analysis reveals potential drug targets in GBM

**Fig. 4:**
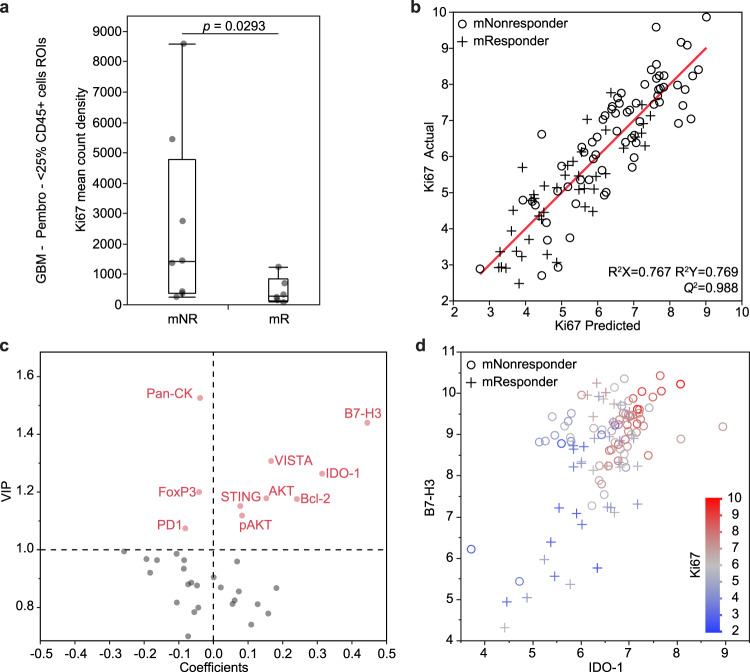
Micro-tumor analysis reveals correlates of tumor proliferation. **a** Box plots of Ki67 expression in the immune-poor regions of interest in GBM samples treated with pembrolizumab (Pembro) (*n* = 8 mNR and 6 mR). The horizontal line in each box represents the median sample value, the ends of the box represent the 25th and 75th percentiles, and the whiskers extend from the ends of the box to the outer most data points. The comparison was made using a two-sided Mann–Whitney *U*-test (*U* = 7). **b**, **c** PLS regression analysis of immune-poor regions of interest with Ki67 as output in GBM samples treated with pembrolizumab. Plot of actual observations versus model predictions of Ki67 expression (**b**). Plot of variable importance in the projection (VIP) versus model coefficients (**c**). The dashed horizontal line represents the VIP > 1.0 cutoff and the dashed vertical line is where the model coefficient is 0. **d** Plot of B7-H3 versus IDO-1, color-coded with Ki67 level (red: high, blue: low). mNR molecular nonresponder, mR molecular responder. Molecular responses are based on a 23-gene molecular signature. Source data are provided as a Source Data file.

As described in the differential analysis above, the tumor-dominant regions of GBM tend to have higher Ki67 expression in mNR tissues (Fig. 4a). To determine the correlates of Ki67, we applied PLSR on the immune-poor regions of GBM, using Ki67 in **Y** and all the other proteins in **X**. As shown in the actual versus predicted plot (Fig. 4b), the optimal six-component model (Supplementary Fig. 7a) captures the variance in the inputs and outputs, and has good predictability (*Q*^2^ = 0.988). Although molecular nonresponder ROIs are found across the full spectrum of Ki67 levels, most of them tend to cluster at high levels of Ki67 (top right corner). In contrast, the molecular responder ROIs tend to cluster at low levels of Ki67 (bottom left corner). The VIP versus coefficient plot reveals that VISTA, AKT, Bcl-2, IDO-1, B7-H3 are positively correlated (coefficient  > 0.1) with Ki67 (Fig. 4c), with IDO-1 and B7-H3 having the strongest correlation in the model (Fig. 4c). In the plot of B7-H3 versus IDO-1, molecular nonresponder ROIs tend to cluster in the top right, and coincide with high tumor proliferation (Fig. 4d).

To test whether Ki67 from the minority immune cells can confound these results, we repeated this analysis at 20% and 30% CD45 cutoff (Supplementary Fig. 7b). We found that the proteins with the highest statistical importance, including VISTA, B7-H3, and IDO-1, remain unchanged. In addition, we found that there is no correlation between Ki67 and immune-cell proportion (r^2^ = 0.07), suggesting that the immune cells contribute to very little of the measured Ki67 signal in the immune poor regions (Supplementary Fig. 7c). Finally, we also carried out a PLSR analysis on the immune poor regions using CD8 as **Y** and all other proteins (including Ki67) in **X**. This analysis confirms that Ki67 is not correlated with CD8 in the immune poor regions (Supplementary Fig. 7d), which is the opposite of the immune rich regions where immune activation is more likely (Fig. 3c, bottom panel). Overall, these analyses suggest that the measured Ki67 mostly comes from tumor cells in the immune-poor regions, and that co-targeting B7-H3 or IDO-1 in neoadjuvant ICB treatment may potentially improve the treatment outcome for patients with recurrent GBM.

### Immune neighbors are associated with treatment response in melanoma

**Fig. 5:**
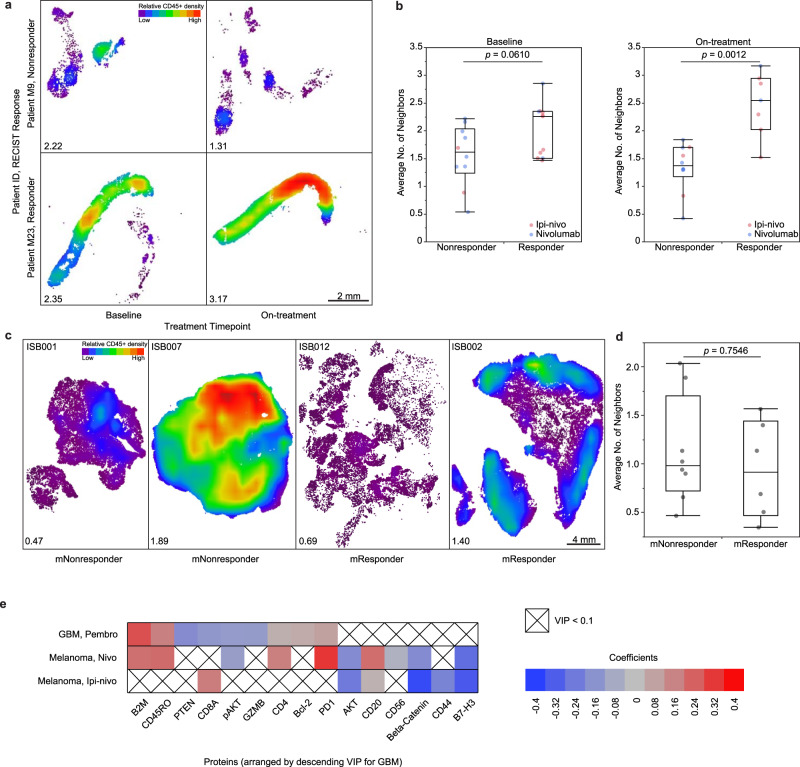
Immune neighbors are associated with treatment response in melanoma. Individual immune cells were identified and mapped out in the tumor, and the number of immune cells adjacent to each immune cell was enumerated and averaged across the tumor. **a** Immune cell density maps of representative melanoma samples from responder and nonresponder at baseline and on-treatment. The average number of immune neighbors for each tissue is indicated at the bottom left. **b** Box plots of the average number of neighbors in melanoma samples at baseline (left) and on-treatment (right) (*n* = 10 nonresponders 11 responders at baseline, *n* = 10 nonresponders and 7 responders on-treatment). Comparisons were made using two-sided Mann–Whitney *U*-tests (*U* = 28 for baseline, *U* = 4 for on-treatment). **c** Immune cell density maps of representative GBM samples from molecular responder and nonresponder. The average number of immune neighbors for each tissue is indicated at the bottom left. **d** Box plots of the average number of neighbors in GBM samples (*n* = 8 mNR and 6 mR). Comparisons were made using two-sided Mann–Whitney U-tests (*U* = 21) **e**, Heat map summarizing PLS regression analyses of immune-rich regions of interest with the average neighbor number of those regions as output. Shown are important (VIP > 1.0) neighbor number predictors that were measured in both melanoma and GBM. Red coloration represents positive coefficients, and blue coloration represents negative coefficients. For box plots in (**b**) and (**d**), the horizontal line in each box represents the median sample value, the ends of the box represent the 25th and 75th percentiles, and the whiskers extend from the ends of the box to the outer most data points. Response for melanoma and GBM are based on response evaluation criteria in solid tumors (RECIST) criteria and a 23-gene molecular signature, respectively. VIP variable importance in the projection. Source data are provided as a Source Data file.

Immune cells often form functional clusters in order to activate tumor-killing programs. To explore this effect, we developed immune neighbor analysis (Fig. 1c), which uses machine-learning-based image analysis to map out the immune cells in the tumor and quantitate the immune cell–cell interaction (Fig. 5)^24,25^. Each immune cell (CD45+) was assigned to a neighbor number, representing the number of immune cells surrounding a given immune cell, and then individual neighbor numbers are averaged across the whole tissue. Overall, the melanoma tissues have a higher mean immune neighbor number than GBM tissues (Fig. 5a and Supplementary Fig. 8), consistent with what is seen in the fluorescent micrographs of these tumors (Fig. 2a, e). Furthermore, in melanoma we found that responders have a higher neighbor number than nonresponders at baseline (*p* = 0.061), and particularly during on-treatment (*p* = 0.001) (Fig. 5b). However, in GBM (Fig. 5c), the mean immune neighbor number does not differentiate mRs from mNRs (*p* = 0.755) (Fig. 5d), suggesting the presence of immunosuppressive CD45+ populations in the tumor microenvironment.

To examine the correlates of immune clustering, we applied PLSR on the immune-rich regions of both tumors, using the immune neighbor number in **Y** and all measured proteins in **X** (Supplementary Fig. 9). The resulting models have excellent predictability for both melanoma treatment arms (*Q*^2^ > 0.98) and modest predictability for GBM (*Q*^2^ = 0.60). The important correlates of immune clustering and their coefficients of the three tumor datasets are summarized in Fig. 5e. In the two monotherapy sets (GBM pembro and melanoma nivo arm), we observed that similar proteins are positively correlated with immune neighbor number, including β2M, CD4, CD45RO, and PD1. Interestingly, we observed that in GBM, immune neighbor number is negatively correlated with CD8 and GZMB, whereas in melanoma (ipi-nivo), immune neighbor number is positively correlated with CD8. This suggests that immune cell–cell interaction in GBM may play a role in tempering CD8 presence and activity.

## Discussion

ICB cancer immunotherapies have exhibited remarkable progress, although pan-cancer success has been elusive. Two extreme examples are melanoma, which serves as a model for cancer immunotherapy response, and GBM, which provides a counter-example. We hypothesized that an in-depth comparison of high-risk melanoma and recurrent GBM tumors, resected from patients following neoadjuvant ICB therapy, might yield insights into improving ICB immunotherapies for treating GBM. To test this hypothesis, we coupled spatially resolved tumor molecular profiling with micro-tumor analysis (Fig. 1b) and immune neighbor analysis (Fig. 1c) to analyze thin FFPE tumor sections resected from 13 patients with recurrent GBM (treated with anti-PD1) and 23 patients with high-risk melanoma (treated with anti-PD1 ± anti-CTLA4). For the melanoma cohorts, patients were classified using standard RECIST criteria of treatment outcome. For the GBM cohort, we used an established^10^ 23-gene transcriptional signature of therapy response to classify the patients as mRs or mNRs (Fig. 1d). This molecular response signature is characterized by upregulation of T cell and interferon-gamma (IFN-γ) genes, and downregulation of cell cycle genes. This downregulation of cell cycling appears to be unique to GBM, as this was not detected in similarly treated melanoma tumors in a different study^26^.

We found major pathophysiological differences between the tumor classes (Fig. 2a, e). Melanoma tumors, especially in responders, exhibit extensive immune-cell rich areas, while immune-cell rich regions within GBM tumors were sparse and contained just a few cells. This was confirmed by quantitative immune neighborhood analysis (Supplementary Fig. 8), which revealed that higher immune cell–cell interaction is predictive of therapy response in melanoma (Fig. 5b), but not in GBM (Fig. 5d). Further, in the immune rich-regions of melanoma, several proteins, including CD19/20, CD8, and CD45RO, are enriched in RECIST responders (Fig. 2d) while for GBM tumors, no measured proteins are enriched in mRs or mNRs (Fig. 2f). Thus, the challenge in resolving the effect of neoadjuvant pembro in recurrent GBM prompted us to develop a data-driven modeling approach based upon two hypotheses. First, the general observation that clinical response to ICB is often accompanied by the infiltration of CD8+ T cells into the tumor tissue^27,28^ suggests that molecular markers of T cell infiltration may also provide markers of therapy response. Second, immune-cell depleted regions within GBM tumors may have immunosuppressive characteristics that are preventing CD8+ T cell infiltration.

We utilized PLS analysis to identify proteins within immune-rich regions that were either predictive of CD8 level, treatment response, or immune cell–cell interaction. This analysis revealed similarities and differences between the two tumor classes (Figs. 3d–g and 5). For example, six proteins, including CD4 and CD45RO, are positive predictors of CD8 in both GBM and at least one of the two melanoma cohorts. We also observed a negative correlation of pSTAT3 with CD8 in both the GBM and melanoma (nivo) tumors, consistent with literature reports that STAT3 signaling may mediate immunosuppression^29–31^. Notably, only in GBM, the macrophage marker CD68 is negatively correlated with CD8. In melanoma, β2M, CD8, and CD19/20 are positive predictors of RECIST response, while AKT and Beta-catenin are negative predictors (Fig. 3e, f and Supplementary Fig. 4). In GBM, PTEN and CD11c are positive predictors of molecular response, while CD44, CD66B, and CD163 are negative predictors (Fig. 3g and Supplementary Fig. 5). Furthermore, immune cell–cell interaction is negatively correlated with CD8 and GZMB in GBM (Fig. 5e), but positively correlated with CD8 in melanoma (ipi-nivo arm).

Together, the differences between the two tumor classes are striking. Unlike melanoma, CD8 presence and MHC class I antigen presentation does not predict therapy response in GBM. Instead, GBM molecular response to ICB appears to depend upon signatures of tumor aggressiveness (pSTAT3, PTEN, and CD44)^11,30,32^ and on the presence of neutrophils and myeloid cells, which have been implicated in glioma progression and treatment resistance^33,34^. In fact, the potential role of CD68+ CD163+ cells in suppressing immune effector activity has been proposed in pembro-treated GBM tumors^12^. Unique also to GBM is the observation that immune cell–cell interaction negatively correlates with CD8 and GZMB. This is consistent with the fact that myeloid-derived suppressive cells (MDSCs), including tumor-associated macrophages (TAMs), can indirectly (via regulatory B cells)^35^ or directly^36^ suppress CD8+ T cell function, including mobility^36^. Macrophage depletion, via colony-stimulating factor 1 receptor (CSF-1R) inhibition, has been shown to increase CD8+ T cell tumor infiltration in tumor-bearing mice, synergizing with anti-PD-1 treatment^36^. Indeed, there exists ongoing phase I trials that use CSF-1R inhibitors (BLZ945 or Cabiralizumab) in combination with anti-PD1 in solid cancers, including GBM (NCT02526017 and NCT02829723)^37,38^.

We further analyzed the immune-poor regions of GBM, since those comprised (by far) the largest area fraction in all GBM tumors. That analysis revealed higher Ki67 expression in mNRs, suggesting tumor cell proliferation (Figs. 2g and 4a). A PLSR analysis revealed that VISTA, AKT, Bcl-2, IDO-1, B7-H3 levels all positively correlate (coefficient >0.1) with Ki67 (Fig. 4c), which are consistent with recent literature. For example, VISTA-deficient murine glioma models are highly resistant to tumor induction^35^. Although the precise role of VISTA is unclear^39^, VISTA-expressing antigen-presenting cells can inhibit T cell proliferation and cytokine production in vitro^40^.

The two strongest positive correlates of tumor growth are B7-H3 and IDO-1 (Fig. 4d), both of which are emerging immunotherapy targets for GBM^41,42^. In fact, mNR patients exhibited the highest levels of B7-H3, IDO-1, and Ki67. IDO-1, which is interferon-inducible^43^, metabolizes tryptophan (Trp) along the l-kynurenine pathway and correlates with decreased patient survival^44^. Reduced Trp levels can cause cell cycle arrest in immune cells, and cause T-cells to become anergic^45^. IDO-1 inhibition plus PD-1 blockade was not successful in a Phase III trial on melanoma patients^46^ but has not been clinically tested for GBM^47^.

B7-H3 strongly associates with Ki67 within immune-poor regions of GBM. It is a CD28-family immune checkpoint that plays roles in T-cell suppression in glioma^48^. In fact, in a recent large study of immunotherapy-related genes in aggressive gliomas, low expression of B7-H3 emerged as the single best predictor of survival^49^. B7-H3 is expressed on immune cells (such as antigen-presenting cells or macrophages) and tumor cells and has an inhibiting influence on both natural killer (NK) cells and cytotoxic T cells^50^. Our data suggest that IDO-1 inhibition, or B7-H3 blockade, when used in combination with anti-PD1, may reduce tumor proliferation in GBM and perhaps improve therapy responses.

The two melanoma treatment cohorts exhibited some differences which should be interpreted with caution due to their small individual cohort sizes^9^. Notable, however, is that responders are enriched for B-cell markers in the nivo arm (Fig. 2b), which prompted some of us to deeply study the involvement of B cells and tertiary lymphoid structures in immunotherapy responses of melanoma and renal cell carcinoma^51^. Further, ipi-nivo nonresponders are enriched for Beta-Catenin (Fig. 2c), which is consistent with recent mouse model reports^52^. Accordingly, indirect^53^ and direct^54^ inhibitors of WNT/beta-catenin signaling are being developed to synergize with checkpoint blockade. Finally, in the analyses of the ipi-nivo arm (Fig. 3b, f), we observed that CD8 is positively correlated with CD45RO and RECIST response. This is consistent with the observation that higher levels of CD45RO+ CD8+ T cells in circulation is associated with better survival in patients with melanoma treated with ipi^55^.

We identified at least three difficulties associated with this study. First, the GBM cohort was treated with neoadjuvant pembro in the off-label setting. Patients with surgically accessible recurrent GBM and who might benefit from this therapy were included in this study. These patients had varying prior treatment and disease histories (Supplementary Table 1), which could introduce inter-patient heterogeneity in the tumors. Second, the number of specimens analyzed here was relatively small (*n* = 8 mNR and 6 mR), which can limit the generalizability of the findings. Third, melanoma and GBM are different in terms of pathophysiology, basal resident immune composition, and route of anti-PD1 entry into the tumor. We attempted to address these challenges with the development of micro-tumor and immune neighbor analyses (Fig. 1b, c), which were able to generate testable hypotheses in both melanoma and GBM, several of which corroborated with literature observations. Future work can take advantage of recent advances in spatial profiling techniques, which are moving towards whole transcriptome analysis and single-cell resolution^56,57^. Such techniques should enable increased levels of quantitation, including cell-type specificity for various protein markers. Nevertheless, the current study was able to identify potential co-targets for enhancing the efficacy of neoadjuvant ICB treatment in recurrent GBM, and permitted comparisons of ICB-treated melanomas and GBMs, which are widely seen as the opposite ends of the spectrum with respect to immunotherapy response.

## Methods

### Patient specimens

Encouraged by the survival benefits of neoadjuvant pembrolizumab treatment in patients with recurrent GBM^10^, 13 patients with recurrent GBM, who did not respond to standard of care therapies, were treated with off-label, off-trial pembrolizumab 200 mg by intravenous infusion 14 ± 5 days before surgical resection. Patients had surgically accessible recurrent GBM, any recurrence, with unequivocal evidence of tumor progression. Patient characteristics included 44% female, mean age of 54.4 years (range 30.2–68.5 years), mean number of recurrences 2.5 (range 1–8), and mean Karnofsky performance status of 84 (range 70–90). Tumor collection and analysis were approved by the Institutional Review Board of the University of California, Los Angeles; all patients provided written informed consent.

In total 18 GBM specimens were collected from the 13 patients. Two patients had successive recurrences and were pre-treated with pembro before each surgical resection. One patient had a multifocal tumor, and specimens were collected from the original tumor location and at a secondary site (Supplementary Table 1). All GBM specimens were analyzed by direct multiplexed mRNA analysis to determine their molecular response, of which 14 specimens were analyzed by spatial protein profiling due to sample availability. The GBM specimens were included in this study as they became available from consecutive patients treated in the off-label setting, without prescreening for a molecular response. In total 39 melanoma specimens were analyzed from 23 patients (Supplementary Table 2), who were enrolled in phase II clinical trial of neoadjuvant ICB (NCT02519322)^9^. All melanoma specimens were analyzed by spatial protein profiling.

### Direct multiplexed mRNA analysis

RNA was isolated from flash-frozen tumor tissue procured from the UCLA Brain Tumor Translational Resource using Tissue Disruption Tubes (Qiagen) and Quick-RNA Mini-prep Plus Kit (Zymo). The nCounter GX analysis system (NanoString) was utilized according to the manufacturer’s directions to quantify RNA expression of 770 genes on the nCounter PanCancer IO360 Panel (NanoString; list of genes available from the manufacturer). NanoString experiments were performed at the UCLA IMT Core/Center for Systems Biomedicine, which is supported by CURE/P30 DK041301. Additional probe information for the 23-gene signature is in Supplementary Data 2.

### Digital spatial profiling

Pre-commercial versions of the GeoMx Digital Spatial Profiler (DSP) (NanoString) were used to quantitate proteins in selected microscopic regions of interest (ROI) in the tumor, as per the methods described in Merritt et al.^16^. Briefly, a multiplexed cocktail of antibodies labeled with either ultraviolet (UV)-cleavable oligonucleotide barcodes or fluorophores was applied to 5 µm-thick formalin-fixed paraformaldehyde-embedded (FFPE) tumor slices. Including control, 31 or 40 oligonucleotide-labeled antibodies were used in the analysis of melanoma or GBM, respectively, with 29 protein targets overlapping between both tumor analyses. The current commercial equivalent of these reagents is the GeoMx Solid Tumor Morp Kit HsP (cat# 121300301) and GeoMx Imm Cell Pro_Hs (cat# 121300101). The targets of the oligonucleotide-labeled antibodies are found in Supplementary Table 3. The spatial protein analysis uses protein targets that have been curated by the vendor for immuno-oncology (I-O) content. These include broad markers of immune cell types (e.g., CD68, CD8, CD4), drug targets being developed within the I-O space (e.g., B7-H3, IDO-1, VISTA), as well as markers for finer immune cell typing (e.g., CD163, CD34, CD45RO). Fluorophore-labeled antibodies against CD45 (melanoma and GBM), S100 (melanoma), GFAP (GBM), along with nuclei stain (SYTO13) were used as morphological markers. After the stained slices were digitally scanned, the morphological markers guided the creation of ROIs for protein profiling. In melanoma, custom ROIs were created for each tumor slice by applying an ImageJ script on thresholded CD45 fluorescent micrographs, facilitating the profiling of CD45+ regions of the tumor. In GBM, geometric ROIs were created across the tissue to profile representative areas of high and low CD45+ cell proportions. The ROIs were then selectively illuminated with UV light to cleave the oligonucleotide barcodes, which were collected by a microcapillary fluidics system and enumerated on the nCounter system. There are several sources of controls built into the methodology of the spatial protein analysis. First, the barcoded antibodies used in this analysis have undergone rigorous testing to ensure specificity, sensitivity, and overall performance, which are in line with guidelines from the Society for Immunotherapy of Cancer^36^. This includes testing in multi-organ tissue microarrays, FFPE cell pellet arrays of positive and negative control cells, and antibody interaction screening to ensure antibodies in the panel do not cross react. Further details and sample data from this process can be found on the vendor website. Second, the antibody panel includes isotype-negative control antibodies that are also conjugated oligo barcode via UV-cleavable linker. Third, a 5-level External RNA Control Consortium (ERCC) spike-in control is included during barcode processing so that batch variations can be corrected during data analysis.

### DSP data processing

Digital counts from barcodes corresponding to proteins were processed in three steps using Microsoft Excel (Redmond, WA). First, raw counts were normalized with ERCC spike-in controls to account for batch and system variation. Second, the normalized counts were subtracted with the appropriate IgG isotype control counts from each ROI to control for nonspecific antibodies; resulting counts that fall below zero were set to zero. Third, the resulting counts were normalized by the ultraviolet-light mask area to yield count density. For inter- and intra-tissue protein expression comparisons, an average count density was first calculated within each tissue compartment (i.e., immune-rich or immune-poor). For example, to calculate Ki67 mean count density shown in Fig. 4a, a Ki67 count density is first calculated for each ROI, which can be categorized as immune poor or immune rich based on the fraction of CD45+cells (Supplementary Fig. 1). Figure 4a compares the immune poor regions of the tumor by averaging the Ki67 count density of the immune-poor ROIs, which yields a Ki67 mean count density for each patient. The mask area division accounts for the varying number of cells that can be captured in different-sized ROIs, and the immune-poor categorization of ROIs ensures that a majority of the Ki67 counts come from non-immune cells.

### PLS analysis

As described above, PLS analysis is designed to extract linear relationships between a matrix **X** of independent variables and a matrix **Y** of dependent variables through the sequential formation of PLS components. PLS components are linear combinations of the original variables formed in a way that maximizes the covariation of **X** with **Y**. After the first component is formed, it is subtracted from the original dataset, and the residual that remains is used to form the next component until the optimal number of components is achieved. In this work, PLS models were constructed in JMP 13.2.1 (SAS, Cary, NC) using the Nonlinear Iterative Partial Least Squares (NIPALS) algorithm, which uses a single dependent variable in **Y**. Before applying the dataset for PLS analyses, the count density was natural logarithm (*x* + 1)-transformed, and inputs to the **X** matrix were standardized (mean-centered and unit variance-scaled). PLS regression (PLSR) analyses used a continuous variable in **Y**, while PLS discriminant analyses (PLS-DA) used a categorical variable in **Y** (i.e., RECIST or molecular response = 1, RECIST or molecular nonresponse = 0). In PLSR analyses samples are excluded if the dependent variable is zero (i.e., signal is indistinguishable from background). A leave-one-out method of cross validation was used to determine the optimal number of components in the model. Here, an individual data element is removed, and the remaining data are fitted with a model, which is used to predict the element that was withheld. This process is repeated until each data element has been withheld once and only once. Summing up the squares of predicted residuals results in the predicted residual sum of squares (PRESS). PRESS is used to calculate the model predictability *Q*^2^, defined as the fraction of the total variation in the **Y** matrix that can be predicted. We also calculate the metrics R^2^X and R^2^Y, which describe how much of the variation in the **X** and **Y** matrix is explained by the model. Every addition of a PLS component contributes to these metrics (R^2^X, R^2^Y, PRESS, and *Q*^2^), and the priority is to minimize PRESS and maximize *Q*^2^. In general, a new component is included if *Q*^2^ is increased or removed if *Q*^2^ is decreased. A van der Voet *T*^2^ statistic test is also used to evaluate whether a model significantly differs from the model with the minimum PRESS. The cumulative R^2^X, R^2^Y, and *Q*^2^ (all have a maximum of 1) are reported in the respective predicted vs actual plots. The regression coefficients and the variable importance of projection (VIP) from the PLS models describe the direction and relative importance of each input, respectively. Typically, VIP values greater than one indicate that a variable is important for predicting the output.

### Machine learning-based image analysis

Image analysis, including cell segmentation and the extraction of single-cell fluorescent intensity (SYTO13 and CD45), size and shape, was performed using a custom pipeline developed in CellProfiler (v 3.1.8)^24,25^. Immune cells were identified by applying classifiers that were generated and trained with object intensity, size, and shape measurements using CellProfiler-Analyst (v 2.2.1). Immune neighbor number, defined as the number of adjacent immune cells surrounding a given immune cell, was enumerated for each immune cell using CellProfiler. Tissue fluorescent micrographs that were out of focus due to tissue delamination or had other imaging defects were excluded from this analysis.

### Statistics

For mRNA quantification data, expression values were normalized using positive and negative controls and housekeeping genes and analyzed using the nSolver analysis software 4.0 (NanoString). For spatial protein quantitation, oligo barcode counts cleaved from the antibodies were normalized to ERCC spike-in controls, background subtracted by IgG isotype control, and normalized to the area of assayed tissue. Mann–Whitney *U*-test (with 95% confidence interval) was used to compare the protein expression, the mean immune neighbor number, or model prediction output between RECIST or molecular responders and nonresponders, between tumor types, or between regions of interest types. Statistical analysis was performed on Graphpad Prism 7 and JMP 13.2.1 32-bit with all *p* values being two-sided.

## Supplementary information

Supplementary Info

Descriptions of Additional Supplementary Files

Dataset 1

Dataset 2

## Data Availability

Spatial protein profiling and immune neighbor datasets for GBM and Melanoma are found in Supplementary Data 1. Source data are available as a Source Data file. High resolution fluorescent micrographs of the tumor specimens are available upon request from the authors due to size limitations. The remaining data are available within the Article, Supplementary Information or Source Data file. Source data are provided with this paper.

## Source data

Source Data File
