# Supplementary material for: Resolution of tissue signatures of therapy response in patients with recurrent GBM treated with neoadjuvant anti-PD1
Source: Nat Commun. 2021 Jun 29;12:4031. doi: 10.1038/s41467-021-24293-4 (PMC8241935; doi:10.1038/s41467-021-24293-4)
Supplement: Supplementary file 1 — Supplementary Info [file 41467_2021_24293_MOESM1_ESM.pdf]

SUPPLEMENTARY INFORMATION

Resolution of tissue signatures of therapy response in patients with recurrent GBM treated with neoadjuvant anti-PD1

Yue Lu[1,†], Alphonsus H. C. Ng[1,†], Frances E. Chow[2], Richard G. Everson[2], Beth A. Helmink[3], Michael T. Tetzlaff[3], Rohit Thakur[3], Jennifer A. Wargo[3], Timothy F. Cloughesy[2], Robert M. Prins[2], and James R. Heath[1,*]

[1]Institute for Systems Biology, Seattle, WA 98109, USA.

[2]Department of Medical and Molecular Pharmacology, David Geffen School of Medicine, University of California, Los Angeles, Los Angeles, CA, USA.

[3]Department of Surgical Oncology, The University of Texas MD Anderson Cancer Center, Houston, TX, USA.

†These authors contributed equally to this work.

*Address correspondence to jim.heath@isbscience.org

Supplementary Table 1: Summary of GBM specimens

Patient ID	Sample ID	Molecular Response	DSP ROIs	Prior treatment	No. of recurrence	Post-Pembro PFS	Post-Pembro OS
G1	ISB001	NR	6	RT, Temodar, Valiparib, DNX-2401, Pembrolizumab	2	49	477
G2	ISB002	R	14	RT, Temodar, Dabrafenib, Trametinib, Pembrolizumab	2	53	53
G1	ISB003	NR	16	RT, Temodar, Valiparib, DNX-2401, Pembrolizumab	4	84	308
G3	ISB004	NR	12	RT, Temodar, Pembrolizumab	1	73	270
G4	ISB005	NR	12	RT, Temodar, Lapatinib, Pembrolizumab	2	46	236
G5	ISB006	R	10	RT, Temodar, Lanreotide, Pembrolizumab	1	125	356
G6	ISB007	NR	16	RT, Temodar, Lapatinib, DNX-2401, Pembrolizumab	2	85	300
G7	ISB008	NR	10	RT, Temodar, PF-06840003, CCNU, Avastin, Carboplatin, Pembrolizumab	5	127	127
G8	ISB009	NR	12	RT, Temodar, Rindopepimut, CCNU, Pembrolizumab, Avastin	8	95	95
G9	ISB010	R	12	RT, Temodar, Avastin, CCNU, DC Vax, Pembrolizumab	2	170	170
G10	ISB011	R	12	RT, Temodar, AMG-596, Pembrolizumab	2	101	159
G11	ISB012	R	12	RT, Temodar, DC Vax, Pembrolizumab	1	68	208
G10	ISB014	R	10	RT, Temodar, AMG-596, Pembrolizumab	2	101	159
G13	ISB015	NR	14	RT, Temodar, Lapatinib, ACP-196, Pembrolizumab	2	109	179
G10	ISB016	R	0	RT, Temodar, AMG-596, Pembrolizumab	4	136	159
G10	ISB017	R	0	RT, Temodar, AMG-596, Pembrolizumab	2	101	159
G14	ISB018	R	0	RT, Temodar, Pembrolizumab	1	413	574
G5	ISB019	R	0	RT, Temodar, Lanreotide, Pembrolizumab	2	166	222

NR, nonresponder; R, responder; RT, radiation therapy; ROIs, regions of interest; DSP, digital spatial profiler

G1 (ISB001 & ISB003) had successive recurrences and was pre-treated with pembrolizumab before both surgeries.

G5 (ISB006 & ISB019) had successive recurrences and was pre-treated with pembrolizumab before both surgeries.

G10 had multifocal tumor. ISB011, ISB014, and ISB017 were from the same surgery after administration of pembrolizumab. ISB011 and ISB017 were from the original tumor location, while ISB014 was at a second tumor site that formed despite treatment to original tumor. ISB016 was from a successive recurrence.

Supplementary Table 2: Summary of melanoma specimens

Patient ID	Sample ID	Timepoint	Treatment Type	RECIST Response	DSP ROIs
M8	MDA001	On-treatment	Ipi-nivo	Nonresponder	6
M9	MDA002	Baseline	Nivolumab	Nonresponder	5
M9	MDA003	On-treatment	Nivolumab	Nonresponder	6
M12	MDA004	On-treatment	Nivolumab	Nonresponder	5
M1	MDA005	Baseline	Ipi-nivo	Responder	6
M2	MDA006	Baseline	Nivolumab	Responder	6
M11	MDA007	On-treatment	Nivolumab	Nonresponder	6
M3	MDA008	Baseline	Ipi-nivo	Nonresponder	5
M5	MDA009	Baseline	Nivolumab	Nonresponder	2
M10	MDA010	Baseline	Ipi-nivo	Responder	6
M6	MDA011	Baseline	Ipi-nivo	Responder	6
M7	MDA012	Baseline	Nivolumab	Nonresponder	6
M10	MDA013	On-treatment	Ipi-nivo	Responder	6
M13	MDA016	Baseline	Ipi-nivo	Responder	6
M12	MDA018	Baseline	Nivolumab	Nonresponder	2
M13	MDA019	On-treatment	Ipi-nivo	Responder	6
M14	MDA020	Baseline	Ipi-nivo	Nonresponder	6
M14	MDA021	On-treatment	Ipi-nivo	Nonresponder	6
M15	MDA022	Baseline	Ipi-nivo	Responder	6
M16	MDA023	Baseline	Nivolumab	Nonresponder	6
M18	MDA024	Baseline	Nivolumab	Nonresponder	4
M15	MDA025	On-treatment	Ipi-nivo	Responder	6
M19	MDA026	Baseline	Ipi-nivo	Responder	5
M19	MDA027	On-treatment	Ipi-nivo	Responder	6
M16	MDA028	On-treatment	Nivolumab	Nonresponder	6
M21	MDA029	Baseline	Nivolumab	Nonresponder	5
M21	MDA030	On-treatment	Nivolumab	Nonresponder	7
M22	MDA031	Baseline	Nivolumab	Nonresponder	5
M22	MDA032	On-treatment	Nivolumab	Nonresponder	6
M11	MDA033	Baseline	Nivolumab	Nonresponder	5
M3	MDA034	On-treatment	Ipi-nivo	Nonresponder	4
M4	MDA035	Baseline	Nivolumab	Responder	5
M4	MDA036	On-treatment	Nivolumab	Responder	5
M23	MDA037	On-treatment	Nivolumab	Responder	6
M5	MDA038	On-treatment	Nivolumab	Nonresponder	6
M17	MDA039	On-treatment	Ipi-nivo	Responder	6
M17	MDA040	Baseline	Ipi-nivo	Responder	2
M20	MDA041	Baseline	Ipi-nivo	Responder	4
M23	MDA042	Baseline	Nivolumab	Responder	6

ROIs, regions of interest; RECIST, response evaluation criteria in solid tumors; DSP, digital spatial profiler

Supplementary Table 3: Digital spatial profiler protein panels

Both	GBM only	Melanoma only
AKT	B7-H4 VTCN1	CD14
B7-H3	CD11c	CD19
Bcl-2	CD163	
Beta-2-Microglobulin	CD34	
Beta-Catenin	CD66B	
CD20	HLA-DR	
CD3	ICOS CD278	
CD4	IDO-1	
CD44	OX40L CD252 TXGP1	
CD45	p-ERK	
CD45RO	STING TMEM173	
CD56		
CD68		
CD8A		
FoxP3		
GZMB		
Histone H3		
Ki67 (8D5)		
MmAb IgG2a		
p-AKT		
Pan-Cytokeratin		
PD1		
PD-L1		
PTEN		
Rabbit IgG		
S6		
STAT3		
STAT3 (phospho Y705)		
VISTA		

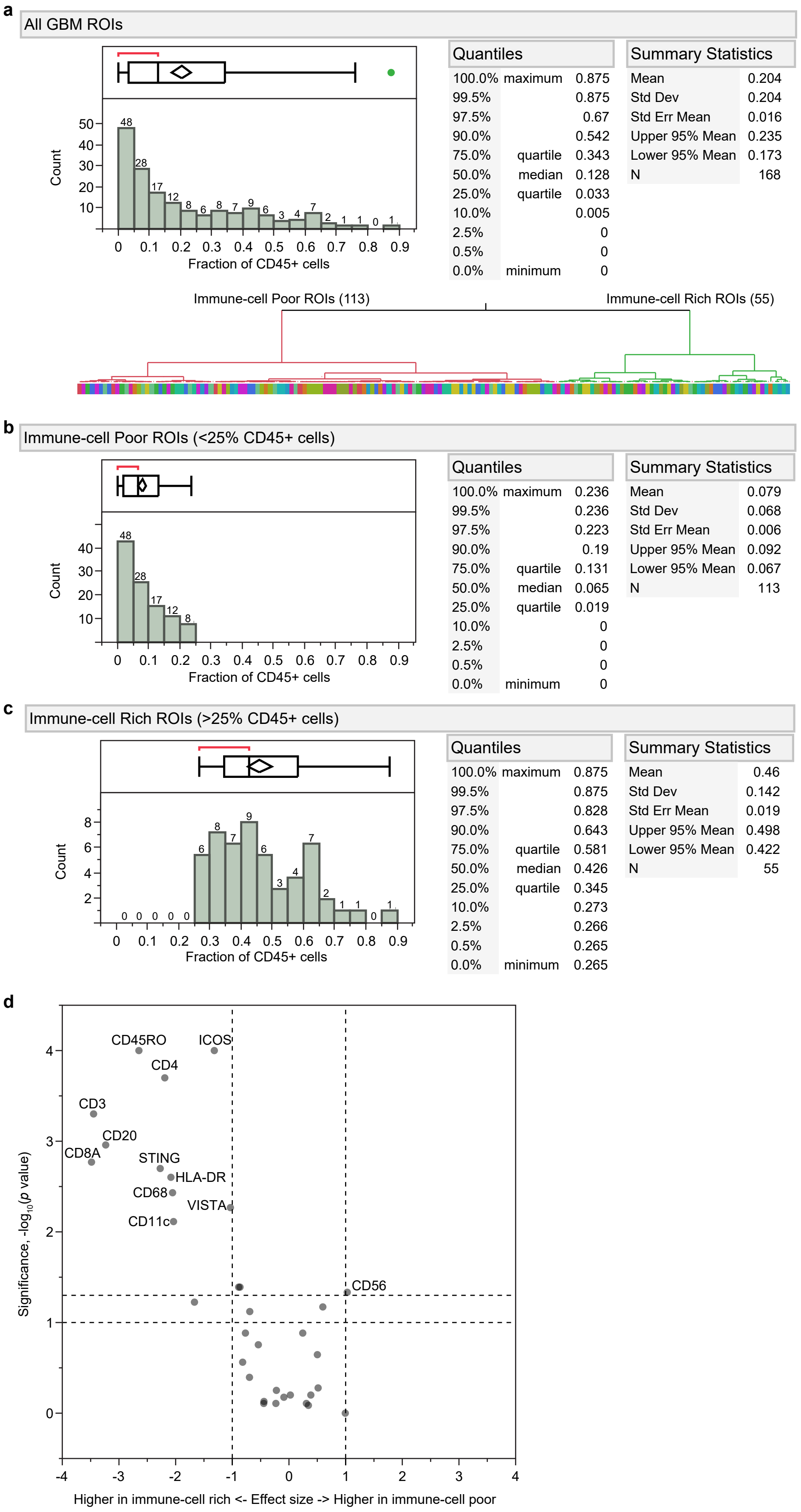
a
All GBM ROIs
Quantiles
100.0% maximum 0.875
99.5% 0.875
97.5% 0.67
90.0% 0.542
75.0% quartile 0.343
50.0% median 0.128
25.0% quartile 0.033
10.0% 0.005
2.5% 0
0.5% 0
0.0% minimum 0
Summary Statistics
Mean 0.204
Std Dev 0.204
Std Err Mean 0.016
Upper 95% Mean 0.235
Lower 95% Mean 0.173
N 168
Count
Fraction of CD45+ cells
48 28 17 12 8 6 8 7 9 6 3 4 7 2 1 1 0 1
Immune-cell Poor ROIs (113)
Immune-cell Rich ROIs (55)
b
Immune-cell Poor ROIs (<25% CD45+ cells)
Quantiles
100.0% maximum 0.236
99.5% 0.236
97.5% 0.223
90.0% 0.19
75.0% quartile 0.131
50.0% median 0.065
25.0% quartile 0.019
10.0% 0
2.5% 0
0.5% 0
0.0% minimum 0
Summary Statistics
Mean 0.079
Std Dev 0.068
Std Err Mean 0.006
Upper 95% Mean 0.092
Lower 95% Mean 0.067
N 113
Count
Fraction of CD45+ cells
48 28 17 12 8
c
Immune-cell Rich ROIs (>25% CD45+ cells)
Quantiles
100.0% maximum 0.875
99.5% 0.875
97.5% 0.828
90.0% 0.643
75.0% quartile 0.581
50.0% median 0.426
25.0% quartile 0.345
10.0% 0.273
2.5% 0.266
0.5% 0.265
0.0% minimum 0.265
Summary Statistics
Mean 0.46
Std Dev 0.142
Std Err Mean 0.019
Upper 95% Mean 0.498
Lower 95% Mean 0.422
N 55
Count
Fraction of CD45+ cells
0 0 0 0 0 6 8 7 9 6 3 4 7 2 1 1 0 1
d
CD45RO
ICOS
CD4
CD3
CD20
CD8A
STING
HLA-DR
CD68
VISTA
CD11c
CD56
Significance, -log10(p value)
Higher in immune-cell rich <- Effect size -> Higher in immune-cell poor

Supplementary Fig. 1: Statistical summary of GBM regions of interest (ROIs) used in digital spatial profiling analysis. a Top: Histogram and statistical summary of all GBM ROIs. Bottom: Dendrogram of hierarchical clustering by Ward's method using the fraction of CD45+ cells as input, color-coded with sample ID. The immune-poor and immune-rich branches are colored in red and green, respectively. **b** and **c** Histogram and statistical summary of ROIs characterized by low immune cell proportions (<25% CD45+ cells) (**b**) and high immune cell proportions (>25% CD45+) (**c**). In the outlier box plots of **a**-**c**, the middle line is the median, the diamond is the mean, the red bracket defines the shortest half of the data (the densest region), and the left and right hinges of the box correspond to the first quartile (Q1) and third quartile (Q3), respectively. The whiskers extend from the box to the furthest point within 1.5 × inter-quartile range (where inter-quartile range equals Q3 minus Q1), and the data beyond the end of the whiskers are outlying points that are plotted individually. **d** Volcano plot of two-sided Mann-Whitney U-test comparisons of the protein expressions between immune-cell rich and immune-cell poor ROIs. Expression of proteins was quantified as average count per area of assayed tissue (n = 14 immune-cell poor and 12 immune-cell rich). The dashed horizontal lines represent the p value cutoffs ($p < 0.10$ and $p < 0.05$) and the dashed vertical lines represent the effect size cutoffs (effect size > |1|). Source data are provided as a Source Data file.

a Melanoma Nivo, immune compartment, CD8 output

Number of factors	Root Mean PRESS		van der Voet T²	Prob > van der Voet T²	Q²	Cumulative Q²	Cumulative R²X	Cumulative R²Y
0	1.021		17.05	<.0001*	-0.04	-0.04	0.000000	0.000000
1	0.511		4.590	0.0270*	0.739	0.739	0.482832	0.770274
2	0.397		0.003	0.9480	0.842	0.959	0.589704	0.883984
3	0.396		0.000	1.0000	0.843	0.994	0.637162	0.916250
4	0.409		0.468	0.5380	0.833	0.999	0.693672	0.926869

b Melanoma Ipi-nivo, immune compartment, CD8 output

Number of factors	Root Mean PRESS		van der Voet T²	Prob > van der Voet T²	Q²	Cumulative Q²	Cumulative R²X	Cumulative R²Y
0	1.022		13.02	<.0001*	-0.04	-0.04	0.000	0.000
1	0.537		0.805	0.3800	0.712	0.712	0.225	0.794
2	0.514		0.485	0.5090	0.736	0.924	0.357	0.854
3	0.505		0.628	0.4380	0.745	0.981	0.463	0.890
4	0.489		0.000	1.0000	0.761	0.995	0.563	0.906
5	0.496		0.075	0.7850	0.754	0.999	0.627	0.921

c GBM Pembro, immune compartment, CD8 output

Number of factors	Root Mean PRESS		van der Voet T²	Prob > van der Voet T²	Q²	Cumulative Q²	Cumulative R²X	Cumulative R²Y
0	1.019		9.906	<.0001*	-0.04	-0.04	0.000	0.000
1	0.810		10.95	<.0001*	0.343	0.343	0.328	0.462
2	0.699		5.018	0.0190*	0.511	0.679	0.530	0.671
3	0.645		2.332	0.1520	0.584	0.867	0.578	0.843
4	0.585		0.844	0.4190	0.658	0.954	0.630	0.893
5	0.556		0.597	0.4720	0.691	0.986	0.668	0.912
6	0.526		0.005	0.9480	0.723	0.996	0.730	0.921
7	0.536		0.276	0.6220	0.713	0.999	0.774	0.932
8	0.527		0.019	0.9050	0.722	1.000	0.810	0.939
9	0.525		0.005	0.9430	0.724	1.000	0.834	0.944
10	0.524		0.000	1.0000	0.725	1.000	0.856	0.950

d

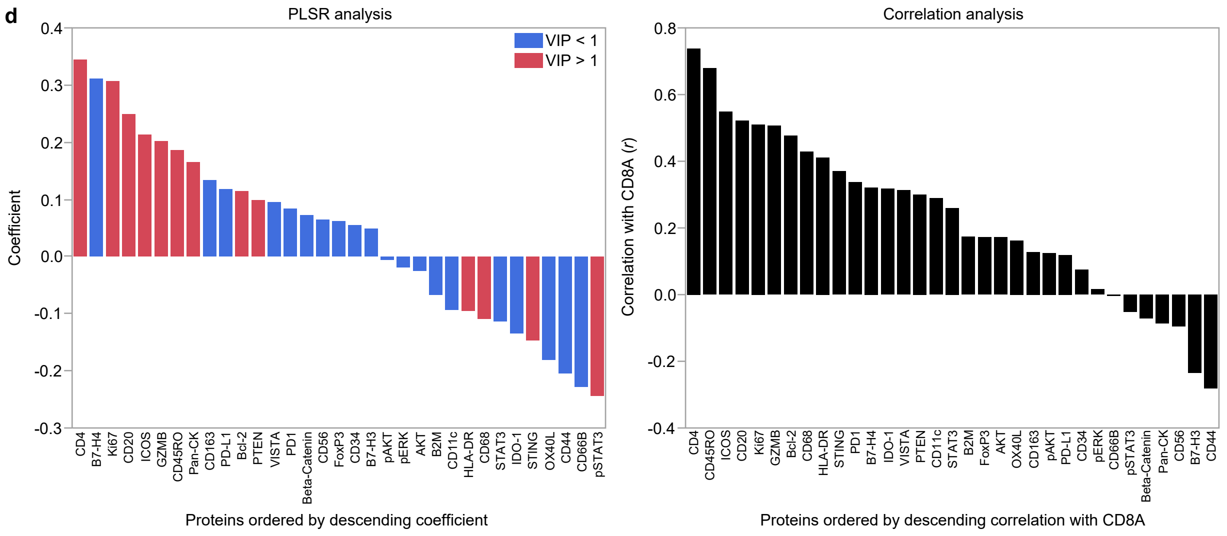

Positive correlation: CD4, Ki67, CD20, ICOS, GZMB, CD45RO, Pan-CK, Bcl-2
Negative correlation: pSTAT3, STING, CD68, HLA-DR

Green: Correlation analysis produced the same sign
Magneta: Correlation analysis produced the different sign

e

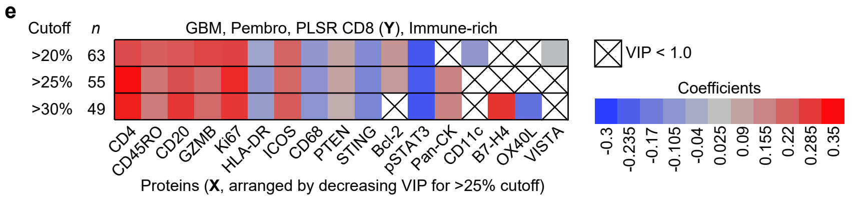

Supplementary Fig. 2: Cross validation summary of PLSR analyses in immune-rich regions of tumors using CD8 in Y and other measured proteins in X and PLSR validation. a-c The optimal number of PLS components is 3, 4, and 6 for melanoma samples treated with nivolumab (nivo) (**a**), melanoma samples treated with ipilimumab plus nivolumab (ipi-nivo) (**b**), and GBM samples treated with pembrolizumab (pembro) (**c**), respectively, as additional PLS components did not improve predictability (Q^2) of model. The optimal number of PLS components and corresponding model metrics are highlighted with a red rectangle. **d** Comparison of PLSR and simple correlation analysis. Left: Bar plot of PLSR analysis with CD8 as output and the other proteins as input. The bars display the value of the model coefficients, and are color coded (based on VIP threshold of 1) to indicate whether the inputs exhibit statistically significant covariation with CD8 (red is significant, blue is insignificant). Right: Bar plot of a correlation analysis with CD8. The bars display the correlation coefficient *r* metric. Bottom: Summary of the positive and negative correlations to CD8 from PLSR analysis. The proteins are color coded to indicate whether the correlation analysis produced an *r* metric with a sign that matches with the PLSR coefficients (green: same, magenta: different). **e** Heat map summarizing PLS regression analyses with CD8 as output of immune-rich GBM ROIs determined by three CD45+ cutoffs (>20%, >25%, and >30%). Shown are important (VIP > 1.0) predictors. Red coloration represents positive coefficients, and blue coloration represents negative coefficients. VIP, variable importance in the projection; ROIs, regions of interest. Source data are provided as a Source Data file.

a Melanoma Nivo, immune compartment, RECIST response catagorical output

Number of factors	Root Mean PRESS		van der Voet T^2	Prob > van der Voet T^2	Q^2	Cumulative Q^2	Cumulative R^2X	Cumulative R^2Y
0	1.019		11.84	<.0001*	-0.04	-0.04	0.000	0.000
1	0.497		0.000	1.0000	0.753	0.753	1.000	0.772

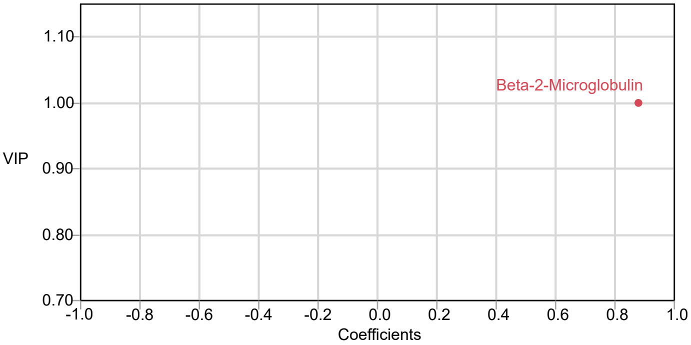

b Melanoma Ipi-nivo, immune compartment, RECIST response catagorical output

Number of factors	Root Mean PRESS		van der Voet T^2	Prob > van der Voet T^2	Q^2	Cumulative Q^2	Cumulative R^2X	Cumulative R^2Y
0	1.022		32.61	<.0001*	-0.04	-0.04	0.000	0.000
1	0.530		0.000	1.0000	0.719	0.719	0.830	0.745
2	0.568		2.116	0.1450	0.678	0.910	0.935	0.755
3	0.562		0.870	0.4040	0.685	0.971	1.000	0.759

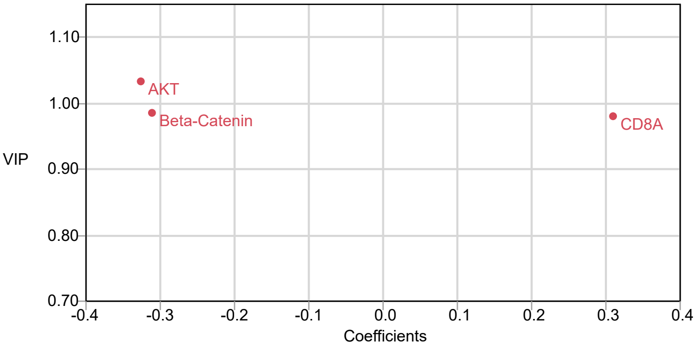

Supplementary Fig. 3: PLS-DA models for individual treatment arms of melanoma using RECIST response as categorical output. a Melanoma samples treated with nivolumab. Top: Cross validation summary with only 1 PLS component as the final model required only one predictor (β2M). Bottom: Plot of VIP versus model coefficient. **b** Melanoma samples treated with ipilimumab plus nivolumab. Top: Cross validation summary indicating that 1 is the optimal number of PLS components. Bottom: Plot of VIP versus model coefficients. The optimal number of PLS components and corresponding model metrics are highlighted with a red rectangle.

a Melanoma both arms combine, immune compartment, RECIST response catagorical output

Number of factors	Root Mean PRESS		van der Voet T^2	Prob > van der Voet T^2	Q^2	Cumulative Q^2	Cumulative R^2X	Cumulative R^2Y
0	1.010		66.51	<.0001*	-0.02	-0.02	0.000	0.000
1	0.582		1.576	0.1980	0.661	0.661	0.512	0.681
2	0.561		0.000	1.0000	0.686	0.893	0.777	0.711
3	0.566		3.107	0.0660	0.680	0.966	0.866	0.712
4	0.565		2.608	0.1130	0.680	0.989	1.000	0.712

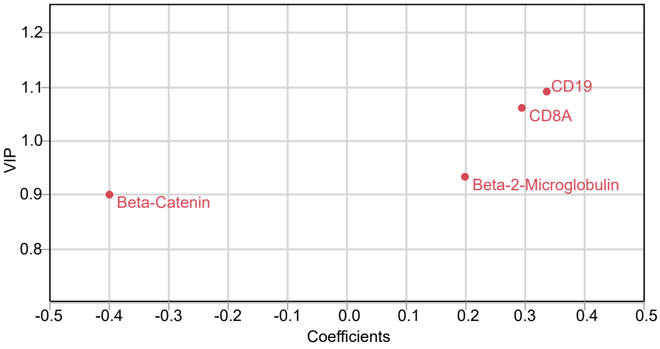

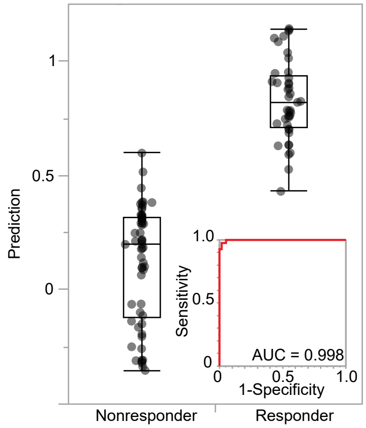

Prediction = 0.122*β2M - 0.168*Beta-Catenin + 0.0717*CD19 + 0.0842*CD8A + 0.139

b

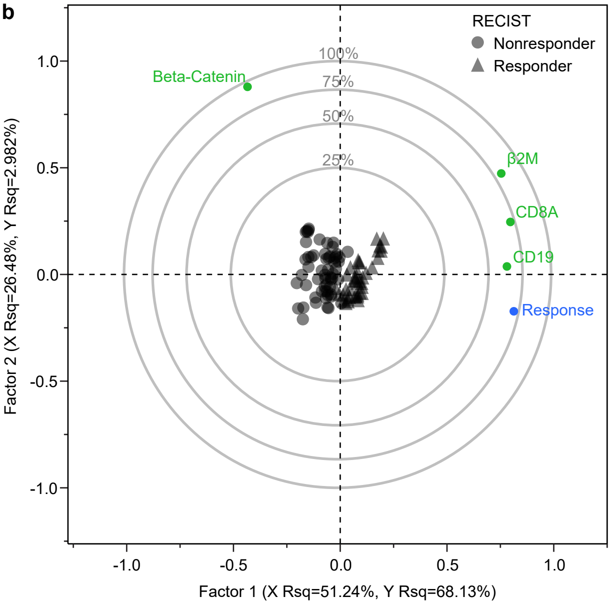

c

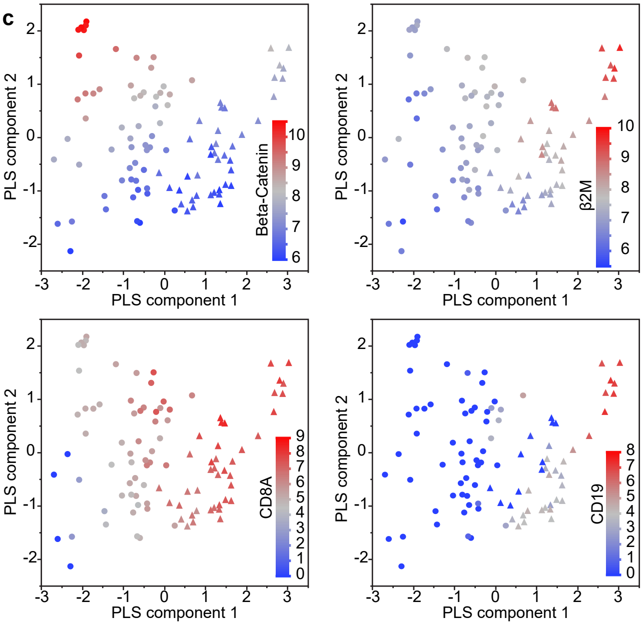

Supplementary Fig. 4: PLS-DA model for combined treatment arms of melanoma using RECIST response as categorical output. **a** Top: Cross validation summary indicating that 2 is the optimal number of PLS components. The corresponding model metrics are highlighted with a red rectangle. Bottom left: Plot of VIP versus model coefficient, with the model prediction equation shown below. Bottom right: Box plots of the final prediction model versus treatment response for individual ROIs. The horizontal line in each box represents the median sample value, the ends of the box represents the 25th and 75th percentiles, and the whiskers extend from the ends of the box to the outer most data points. Inset, area under the receiver operating characteristics curve (AUC) (n = 58 nonresponders and 41 responders). **b** Correlation-loading plot showing the relationship between the final predictors (green circles) and the response (blue circle), and the relative contributions of the predictors to each PLS component. The proximity of the predictors to the response indicate the strength of their correlation. The relative position of each ROI in PLS component space is shown near the origin (triangle: responder, circle: nonresponder). **c** Plots of ROI positions in PLS component space color-coded with the level of each proteins (red: high, blue: low). Response for melanoma is based on RECIST criteria. ROI, region of interest. Source data are provided as a Source Data file.

a GBM Pembro, immune-rich compartment, molecular response catagorical output

Number of factors	Root Mean PRESS		van der Voet T^2	Prob > van der Voet T^2	Q^2	Cumulative Q^2	Cumulative R^2X	Cumulative R^2Y
0	1.019		20.72	<.0001*	-0.04	-0.04	0.000	0.000
1	0.741		0.662	0.4230	0.451	0.451	0.263	0.532
2	0.716		0.000	1.0000	0.487	0.719	0.500	0.584
3	0.717		0.006	0.9300	0.486	0.855	0.712	0.591

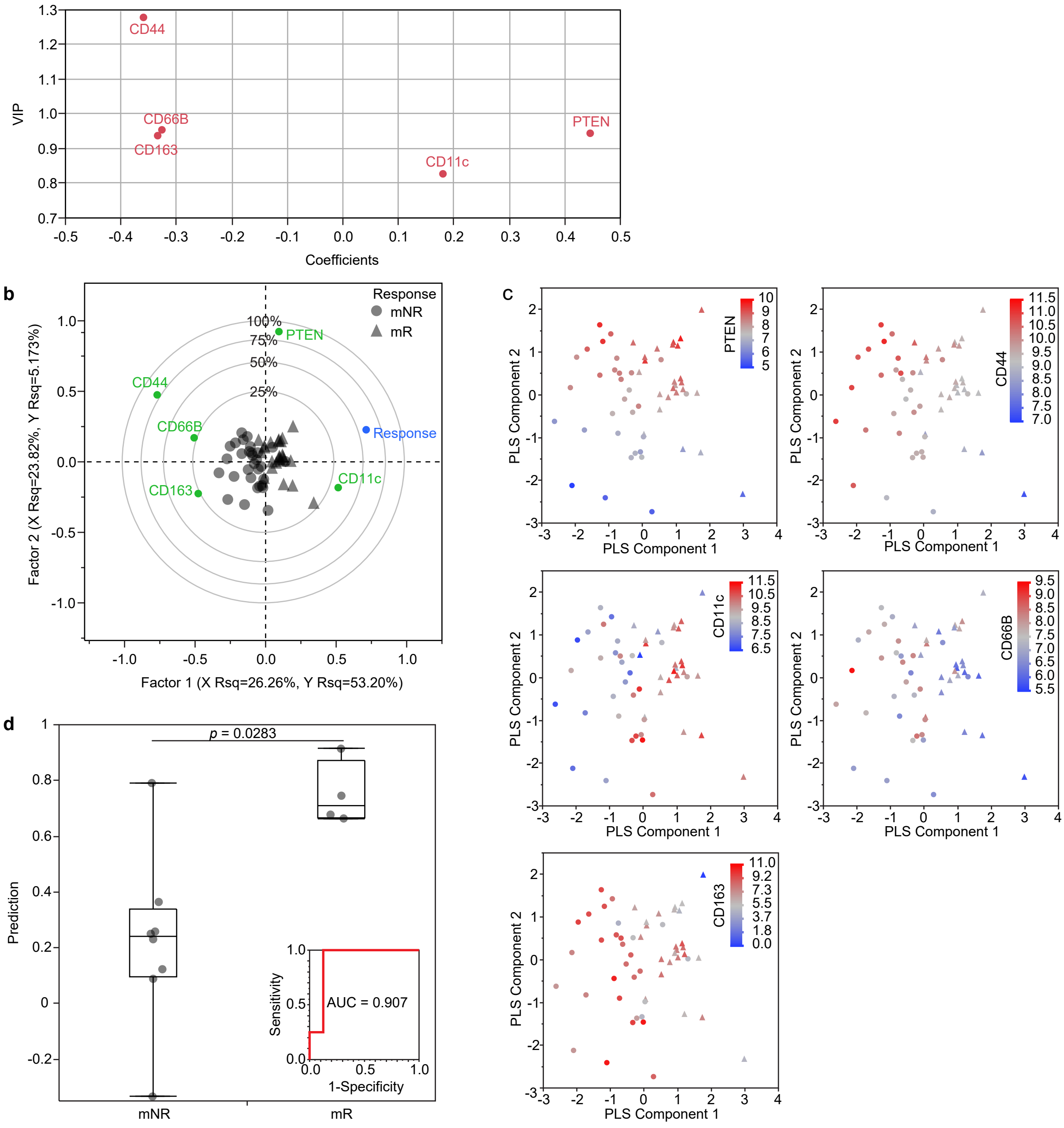

Supplementary Fig. 5: PLS-DA model for immune-rich regions of GBM using molecular response as categorical output. a Top: Cross validation summary indicating that 2 is the optimal number of PLS components. The corresponding model metrics are highlighted with a red rectangle. Bottom: Plot of VIP versus model coefficient. **b** Correlation-loading plot showing the final predictors (green circles) and the molecular response (blue circle). The relative position of each ROI in PLS component space is shown near the origin (triangle: mR, circle: mNR). **c** Plots of ROI positions in PLS component space color-coded with the level of each proteins (red: high, blue: low). **d** The prediction formula was applied at the tissue level, which enabled accurate classification of mR and mNR tissues (AUC = 0.907). Shown are box plots of the final prediction model versus molecular response in the immune-rich regions of GBM samples treated with pembrolizumab (n = 8 mNR and 4 mR). The horizontal line in each box represents the median sample value, the ends of the box represents the 25th and 75th percentiles, and the whiskers extend from the ends of the box to the outer most data points. Inset, area under the receiver operating characteristics curve (AUC). Comparison was made using a two-sided Mann-Whitney U-test (U = 3). Response for GBM is based on a 23-gene molecular signature. ROI, region of interest. Source data are provided as a Source Data file.

GBM Pembro, immune-poor compartment, molecular response catagorical output

Number of factors	Root Mean PRESS		van der Voet T²	Prob > van der Voet T²	Q²	Cumulative Q²	Cumulative R²X	Cumulative R²Y
0	1.009		8.937	0.0010*	-0.02	-0.02	0.000	0.000
1	0.855		0.342	0.5650	0.269	0.269	0.479	0.321
2	0.843		0.000	1.0000	0.290	0.481	0.771	0.356
3	0.858		1.830	0.1550	0.264	0.618	0.876	0.358

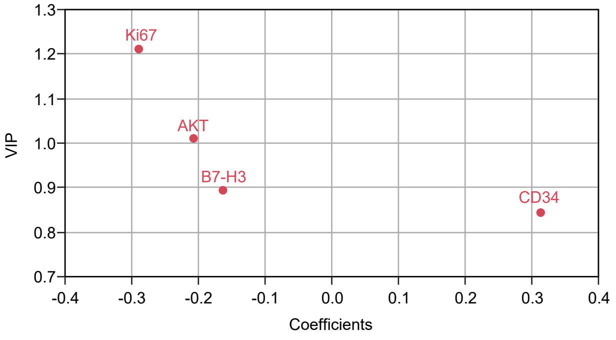

Prediction = -0.0888*AKT - 0.0635*B7-H3 + 0.150*CD34 - 0.0763*Ki67 + 1.08

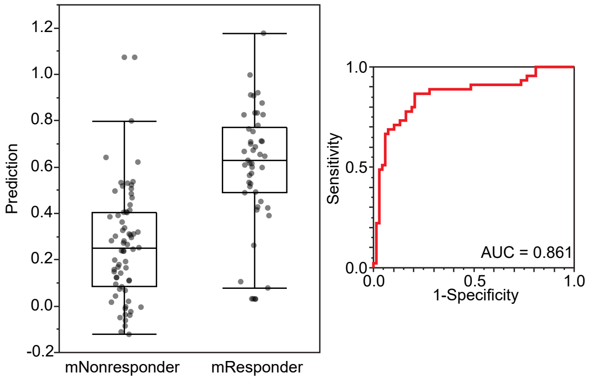

Supplementary Fig. 6: PLS-DA model for immune-poor regions of GBM using molecular response as categorical output. Top: Cross validation summary indicating that 2 is the optimal number of PLS components. The corresponding model metrics are highlighted with a red rectangle. Middle: Plot of VIP versus model coefficient, with the model prediction equation shown below. Bottom left: Outlier box plots of the final prediction model versus molecular response for individual ROIs (*n* = 68 mNR and 45 mR). The horizontal line in each box represents the median sample value, and the ends of the box represent the first quartile (Q1) and third quartile (Q3). The whiskers extend from the box to the furthest point within 1.5 × inter-quartile range (where inter-quartile range equals Q3 minus Q1), and the data beyond the end of the whiskers are outlying points that are plotted individually. Bottom right: area under the receiver operating characteristics curve (AUC). Response for GBM is based on a 23-gene molecular signature. ROIs, regions of interest. Source data are provided as a Source Data file.

a GBM Pembro, immune-poor compartment, Ki67 output

Number of factors	Root Mean PRESS		van der Voet T²	Prob > van der Voet T²	Q²	Cumulative Q²	Cumulative R²X	Cumulative R²Y
0	1.009		22.55	<.0001*	-0.02	-0.02	0.000	0.000
1	0.811		6.268	0.0060*	0.342	0.342	0.461	0.384
2	0.681		0.226	0.6450	0.537	0.695	0.537	0.637
3	0.666		0.036	0.8350	0.556	0.865	0.583	0.707
4	0.678		0.624	0.4380	0.540	0.938	0.643	0.748
5	0.663		0.026	0.8890	0.560	0.973	0.729	0.761
6	0.661		0.000	1.0000	0.563	0.988	0.767	0.769
7	0.675		1.519	0.2300	0.545	0.995	0.802	0.777

b

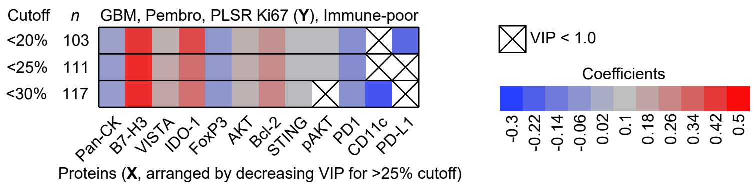

c

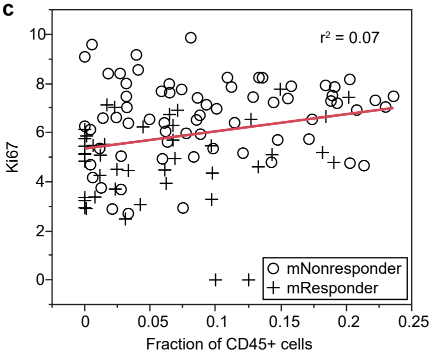

d

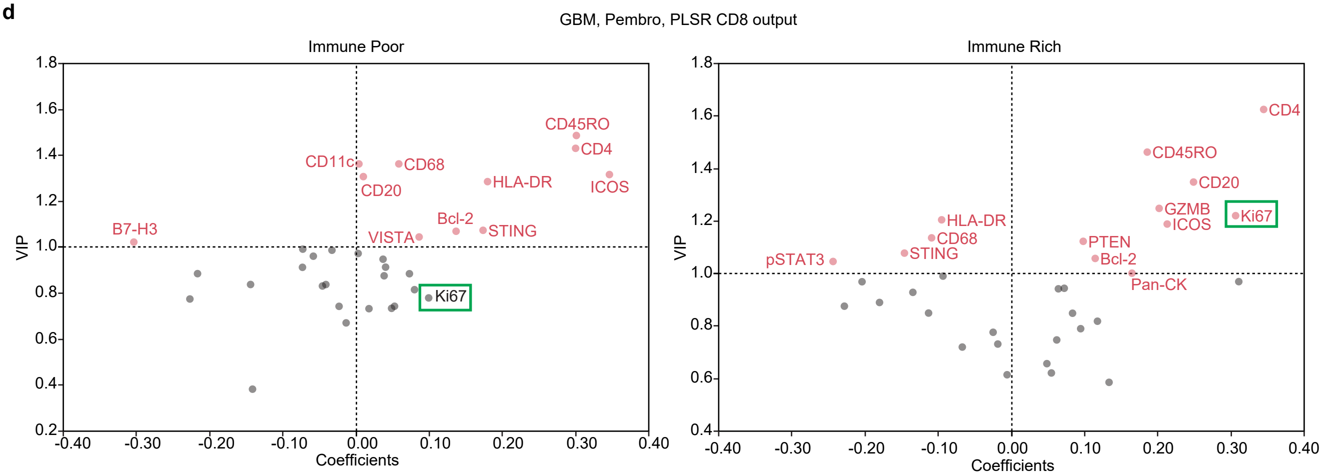

Supplementary Fig. 7: Cross validation summary of PLSR analysis in immune-poor regions of GBM treated with pembrolizumab using Ki67 in Y and other measured proteins in X and PLSR validation. a The optimal number of PLS components is 6 as additional PLS components did not improve predictability (Q^2) of model. The optimal number of PLS components and corresponding model metrics are highlighted with a red rectangle. **b** Heat map summarizing PLS regression analyses with Ki67 as output of immune-poor GBM ROIs determined by three CD45+ cutoffs (<20%, <25%, and <30%). Shown are important (VIP > 1.0) predictors. Red coloration represents positive coefficients, and blue coloration represents negative coefficients. **c** Plot of Ki67 versus fraction of CD45+ cells. Red line is a linear regression of the data and r^2 is the coefficient of determination for the regression. **d** Plots of VIP versus model coefficients for PLSR analyses with CD8 as output in the immune-poor (left) and immune-rich (right) regions of GBM. The dashed horizontal line represents the VIP > 1.0 cutoff and dashed vertical line is where the model coefficient is 0. Ki67 is highlighted with a green rectangle. Response for GBM is based on a 23-gene molecular signature. ROIs, regions of interest. Source data are provided as a Source Data file.

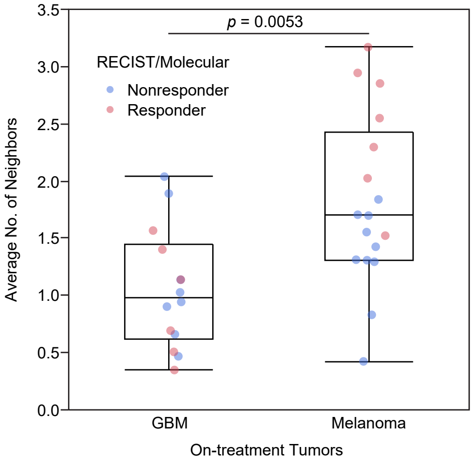

p = 0.0053
RECIST/Molecular
Nonresponder
Responder
Average No. of Neighbors
3.5
3.0
2.5
2.0
1.5
1.0
0.5
0.0
GBM
Melanoma
On-treatment Tumors

Supplementary Fig. 8: Comparison of neighbor numbers in on-treatment GBM and melanoma. Box plots of the average number of neighbors in GBM and melanoma color coded with RECIST or molecular response (n = 14 GBM and 17 melanoma). The horizontal line in each box represents the median sample value, the ends of the box represents the 25th and 75th percentiles, and the whiskers extend from the ends of the box to the outer most data points. Comparison was made using a two-sided Mann-Whitney U-test (U = 50). Response for melanoma and GBM are based on RECIST criteria and a 23-gene molecular signature, respectively. Source data are provided as a Source Data file.

a Melanoma Nivo, immune compartment, Average No. of neighbor output

Number of factors	Root Mean PRESS	van der Voet T²	Prob > van der Voet T²	Q²	Cumulative Q²	Cumulative R²X	Cumulative R²Y
0	1.019	14.24	<.0001 *	-0.04	-0.04	0.000	0.000
1	0.782	10.74	<.0001 *	0.388	0.388	0.463	0.456
2	0.628	3.419	0.0550	0.605	0.759	0.586	0.706
3	0.557	1.971	0.1690	0.690	0.925	0.631	0.824
4	0.532	0.000	1.0000	0.717	0.979	0.692	0.845
5	0.534	0.021	0.8810	0.715	0.994	0.753	0.856

b Melanoma Ipi-nivo, immune compartment, Average No. of neighbor output

Number of factors	Root Mean PRESS	van der Voet T²	Prob > van der Voet T²	Q²	Cumulative Q²	Cumulative R²X	Cumulative R²Y
0	1.022	17.65	<.0001 *	-0.04	-0.04	0.000	0.000
1	0.658	9.121	<.0001 *	0.567	0.567	0.212	0.685
2	0.570	3.983	0.0330 *	0.675	0.859	0.374	0.810
3	0.478	0.522	0.4910	0.771	0.968	0.470	0.891
4	0.457	0.032	0.8820	0.791	0.993	0.600	0.904
5	0.457	0.093	0.7520	0.791	0.999	0.671	0.918
6	0.452	0.000	1.0000	0.796	1.000	0.766	0.925
7	0.460	0.138	0.7330	0.788	1.000	0.818	0.930

c GBM pembro, immune compartment, Average No. of neighbor output

Number of factors	Root Mean PRESS	van der Voet T²	Prob > van der Voet T²	Q²	Cumulative Q²	Cumulative R²X	Cumulative R²Y
0	1.019	3.934	0.0450*	-0.04	-0.04	0.000	0.000
1	0.902	1.192	0.3020	0.186	0.186	0.373	0.304
2	0.850	0.276	0.6170	0.277	0.411	0.489	0.515
3	0.830	0.000	1.0000	0.311	0.595	0.582	0.614
4	0.843	0.121	0.7530	0.289	0.712	0.654	0.672

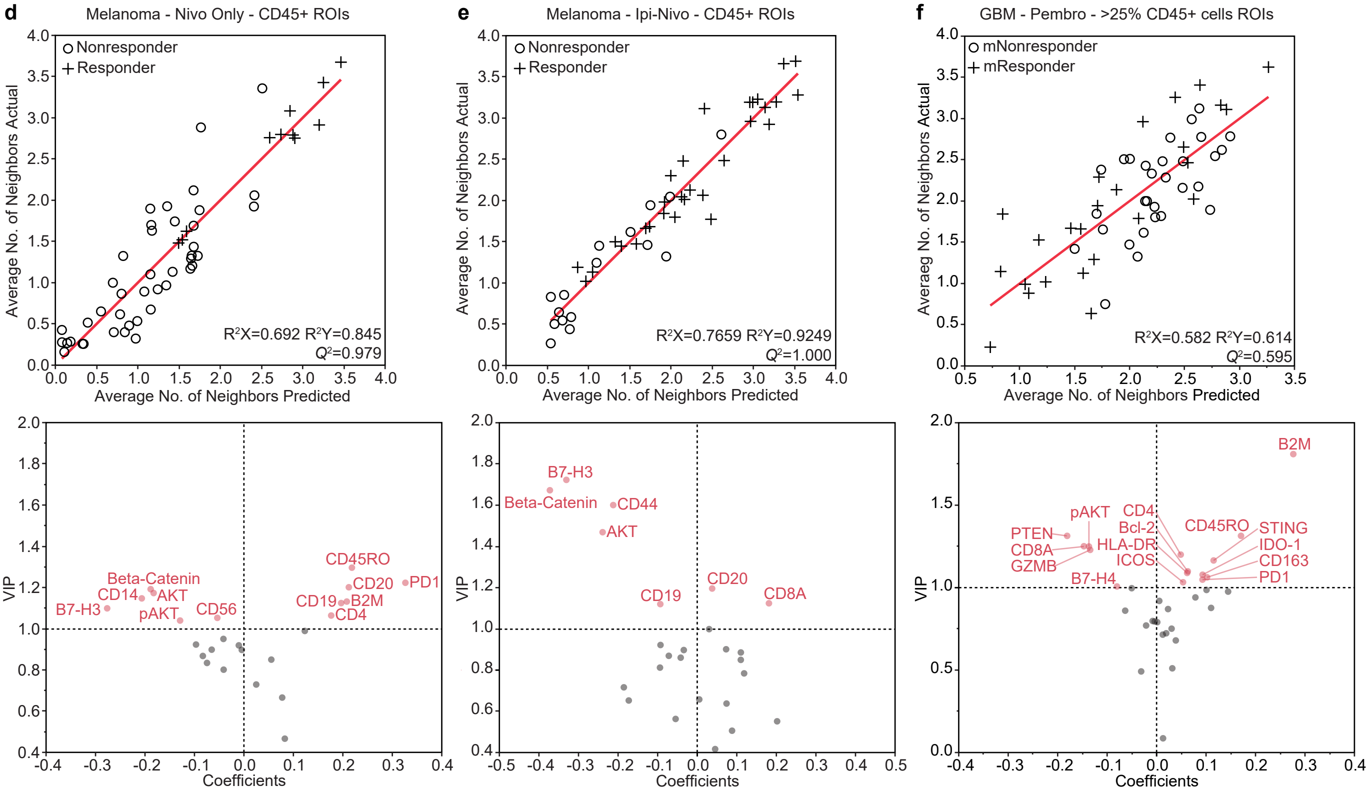

Supplementary Fig. 9: PLSR analyses in immune-rich regions of tumors using average neighbor in Y and other measured proteins in X. a-c The optimal number of PLS components is 4, 6, and 3 for melanoma samples treated with nivolumab (**a**), melanoma samples treated with ipilimumab plus nivolumab (**b**), and GBM samples treated with pembrolizumab (**c**), respectively. The optimal number of PLS components and corresponding model metrics are highlighted with a red rectangle. **d-f** Plots of actual vs. predicted (top panel) and VIP vs. coefficients (bottom panel) for melanoma samples treated with nivolumab (**d**), melanoma samples treated with ipilimumab plus nivolumab (**e**), and GBM samples treated with pembrolizumab (**f**). The dashed horizontal line represents the VIP > 1.0 cutoff and dashed vertical line is where the model coefficient is 0. Response for melanoma and GBM are based on RECIST criteria and a 23-gene molecular signature, respectively. Source data are provided as a Source Data file.
