# Supplementary material for: Resolution of tissue signatures of therapy response in patients with recurrent GBM treated with neoadjuvant anti-PD1
Source: Nat Commun. 2021 Jun 29;12:4031. doi: 10.1038/s41467-021-24293-4 (PMC8241935; doi:10.1038/s41467-021-24293-4)
Supplement: Supplementary file 2 — Descriptions of Additional Supplementary Files [file 41467_2021_24293_MOESM2_ESM.pdf]

Descriptions of Additional Supplementary Files

Supplementary Data 1
Description: Spatial protein profiling and immune neighbor datasets for GBM and Melanoma.

Supplementary Data 2
Description: Probe information for the 23-gene molecular signature.
